# Correlation of age of onset and clinical severity in Niemann–Pick disease type C1 with lysosomal abnormalities and gene expression

**DOI:** 10.1038/s41598-022-06112-y

**Published:** 2022-02-09

**Authors:** Laura L. Baxter, Dawn E. Watkins-Chow, Nicholas L. Johnson, Nicole Y. Farhat, Frances M. Platt, Ryan K. Dale, Forbes D. Porter, William J. Pavan, Jorge L. Rodriguez-Gil

**Affiliations:** 1 Genomics, Development and Disease Section, Genetic Disease Research Branch, National Human Genome Research Institute, National Institutes of Health, Bethesda, MD USA; 2 Bioinformatics and Scientific Programming Core, Eunice Kennedy Shriver National Institute of Child Health and Human Development, National Institutes of Health, Bethesda, MD USA; 3 Division of Translational Medicine, Eunice Kennedy Shriver National Institute of Child Health and Human Development, National Institutes of Health, Bethesda, MD USA; 4 Department of Pharmacology, University of Oxford, Oxford, UK; 5 Division of Medical Genetics, Stanford University School of Medicine, Stanford, CA USA; 6 Department of Pediatrics, Stanford University School of Medicine, Stanford, CA USA

**Keywords:** Medical genetics, Diseases of the nervous system, Endocrine system and metabolic diseases, Medical research

## Abstract

Niemann–Pick disease type C1 (NPC1) is a rare, prematurely fatal lysosomal storage disorder which exhibits highly variable severity and disease progression as well as a wide-ranging age of onset, from perinatal stages to adulthood. This heterogeneity has made it difficult to obtain prompt diagnosis and to predict disease course. In addition, small NPC1 patient sample sizes have been a limiting factor in acquiring genome-wide transcriptome data. In this study, primary fibroblasts from an extensive cohort of 41 NPC1 patients were used to validate our previous findings that the lysosomal quantitative probe LysoTracker can be used as a predictor for age of onset and disease severity. We also examined the correlation between these clinical parameters and RNA expression data from primary fibroblasts and identified a set of genes that were significantly associated with lysosomal defects or age of onset, in particular neurological symptom onset. Hierarchical clustering showed that these genes exhibited distinct expression patterns among patient subgroups. This study is the first to collect transcriptomic data on such a large scale in correlation with clinical and cellular phenotypes, providing a rich genomic resource to address NPC1 clinical heterogeneity and discover potential biomarkers, disease modifiers, or therapeutic targets.

## Introduction

Niemann–Pick disease type C1 (NPC1) is a rare, recessive, lysosomal storage disorder associated with neurodegenerative phenotypes as well as hepatomegaly and splenomegaly (OMIM #257220). Clinical manifestations of NPC1 disease vary and often correlate with age of onset, which can occur from the prenatal period to well into adulthood^1–3^. Biallelic mutation of *NPC1,* which encodes the lysosomal transmembrane protein NPC1, is causative for this disease^4^. NPC1 performs an essential role in lysosomal cholesterol trafficking, and loss of function of the *NPC1* gene causes accumulation of unesterified cholesterol in late endosomes/lysosomes as well as abnormally high glycosphingolipid levels, especially in the central nervous system (CNS)^5–9^. Loss of NPC1 function also results in accumulation of lipids, which gives rise to enlarged, lipid-laden macrophages that are present in peripheral tissues, notably lung, liver and spleen^10–15^.

Prenatal NPC1 disease is rare and has a very poor prognosis, with splenomegaly, hepatomegaly, and fetal ascites seen as the most common phenotypes^16^. In neonatal NPC1 patients, cholestatic jaundice and liver dysfunction occur frequently; while some neonatal patients exhibit transient hepatosplenomegaly that resolves by 4 months of age, up to 10% of others exhibit rapidly progressing liver dysfunction that is fatal^1,17^. While these phenotypes can lead to neonatal diagnosis, diagnosis is frequently missed until later ages, when the neurological abnormalities that are the classical primary presentation in NPC1 disease arise. Neurological phenotypes can present during infantile ages as symptoms of hypotonia and motor delay, which are frequently identified due to increased clumsiness and learning difficulties in school^1,18^. In the juvenile stage, other neurological signs often arise, such as gait disturbance and ataxia accompanied by fine-motor skill impairment; these are related to cerebellar neurodegeneration, in particular Purkinje neuron loss^19^. Adult onset NPC1 disease can present with more common psychiatric manifestations such as cognitive decline, bipolar disorder or schizophrenia, often resulting in missed or delayed diagnosis^20–23^. Previous reports have shown that the age of onset, in particular neurological symptom onset, often correlates with lifespan/disease progression in NPC1 patients^1,24^. Therefore, characterizing a patient’s age of neurological onset is essential for predicting disease course as well as identifying candidates who would benefit from earlier treatment interventions. In addition, a method that quantitatively scores neurological severity in NPC1 disease across 17 clinical phenotype domains and can be adjusted to age of onset has recently been developed, and this system can be used to monitor neurological disease status and subsequent progression^25,26^.

Currently, there are very limited treatment options available for NPC disease, and most potential therapies are undergoing clinical trials^27^. Of note, these treatments primarily target the CNS. The oligosaccharide derivative 2-hydroxypropyl-β-cyclodextrin (HPβCD) has been shown to delay disease progression and increase lifespan in the *Npc1*^*m1N*^ mouse model of NPC1 disease^28–33^. A recently completed phase 1-2a clinical trial (NCT01747135, ClinicalTrials.gov) extended these results to individuals with NPC1 disease and suggested that intrathecal treatment with HPβCD slowed disease progression^26^. However, a subsequent 12-month, mock-controlled clinical trial has not demonstrated efficacy (NCT02534844, ClinicalTrials.gov). The glucosyl ceramide synthase inhibitor *N*-butyldeoxynojirimycin (miglustat, Zavesca) is correlated with reduced glycosphingolipid levels, stabilized neurological phenotypes, and significantly reduced mortality risk in NPC1^34–37^, and is the only treatment that is approved for NPC disease worldwide^38^. However, the FDA has not approved its use for NPC disease, thus miglustat is limited to off-label use for NPC in the United States. Recent studies have proposed that the heat-shock protein co-inducer arimoclomol may have potential therapeutic benefit for neurological disorders, including NPC1^39^ (NCT02612129, ClinicalTrials.gov). Several studies using these potential therapies suggest that treatment at early stages of the disease may be more beneficial than at later stages^29,37,40,41^. Therefore, the ability to predict NPC1 disease severity, progression, and individual treatment response could greatly improve disease management.

Previously, we found that LysoTracker Red (LysoTracker) staining in fibroblasts showed promise as a marker that correlated with the age of onset of any NPC1-associated symptom, and showed the highest correlation with age of onset of neurological symptoms^42^. LysoTracker staining can be used as a cellular biomarker in circulating B cells of NPC1 patients to quantitate the enlargement of acidic, endolysosomal storage compartments that occurs because of accumulated unesterified cholesterol^43^. LysoTracker has also been used in the lysosomal storage disorder Gaucher disease to identify abnormally large endolysosomal compartments^44^. In addition, further understanding of the gene expression changes that arise in NPC1 disease could improve prediction of disease course, as this knowledge could lead to discovery of NPC1 genetic modifiers, biomarkers, and novel pathways involved in disease progression. However, as is the case for other orphan disorders, the small NPC1 patient population has previously been a limiting factor in gathering comprehensive genomic and transcriptomic datasets. In this study, we analyzed clinical data for 41 patients enrolled in an NPC1 natural history study along with quantitative LysoTracker staining and RNA gene expression of primary fibroblasts from these patients. Our results validate and extend previous data showing that LysoTracker levels correlate with age of onset, with the highest correlation to neurological onset, and also correlate with disease severity. Additionally, we used genome-wide expression analyses to identify 127 genes that significantly correlated with LysoTracker staining, disease severity, or age of onset. Hierarchical clustering analysis of these significantly correlated genes indicated distinct gene expression profiles across the cohort, suggesting genes and pathways that may contribute to phenotypic heterogeneity and varied response to HPβCD treatment. This comprehensive dataset provides an important resource to guide future studies on NPC disease biomarker validation and modifier identification, thus furthering our understanding of NPC1 disease biology in the setting of genetic and phenotypic heterogeneity.

## Results

### Quantitative LysoTracker measurements in NPC1 fibroblasts are reproducible

As part of an NPC1 natural history study at the National Institutes of Health (NCT00344331, Clinical Trials.gov), primary skin fibroblasts were collected by skin biopsy from 41 individuals with NPC1. This expanded a fibroblast set from an NPC1 patient cohort that was previously published by our group^42^ and included 24 of the 27 patients in the previous study along with 17 additional patients. The demographics of these 41 individuals are described in Table 1.

**Table 1:** NPC1 patient demographics.

Age of onset (years)	Age of first neurological symptom (years)	Age-adjusted neurological severity score	LysoTracker levels (fold change)	Presenting symptom	First neurological symptom	Sex	Pathogenic DNA variants	Predicted protein changes
N/A	N/A	0.1	9.7	None	None reported	M*	c.410C>T, c.2000C>T	p.T137M, p.S667L
− 0.4**	N/A	0.0	43.1	Hyperecholic intestines, IUGR	None reported	F	c.3182T>C, c.1628C>T	p.I1061T, p.P543L
0	N/A	2.0	30.9	Splenomegaly	None reported	M	c.3182T>C, c.3182T>C	p.I1061T, p.I1061T
0	N/A	0.0	38.3	Jaundice, liver failure^†^	None reported	M	c.3182T>C, c.681T>G	p.I1061T, p.C227W
0.1	N/A	2.4	36.7	Jaundice	None reported	M	c.3182T>C, c.3107C>T	p.I1061T, p.T1036M
0.5	N/A	0.1	9.9	Splenomegaly	None reported	F	c.2932C>T, c.3246-2A>G	p.R978C, (predicted abnormal splicing)
1	N/A	0.1	7.1	Hepatosplenomegaly	None reported	F*	c.665A>G, c.1402T>G	p.N222S, p.C468G
2	N/A	0.1	7.8	Splenomegaly	None reported	F*	c.665A>G, c.1402T>G	p.N222S, p.C468G
− 0.4**	1.2	0.8	24.1	Fetal ascites	Not walking at 14 months	F	c.3182T>C, c.3182T>C	p.I1061T, p.I1061T
0	1	8.0	66.6	Jaundice	Developmental plateau	M	c.3565_3566insG, 2008_2011delTGCT	p.E1189Gfs*69, p.C670Pfs*12
0	1.5	5.1	47.9	Hepatosplenomegaly	Loss of gross motor skills, dysphagia	M	c.2978delG, c.3591+4delA	p.G993Efs*4, (possible splicing effect)
0	3	2.2	17.9	Jaundice, splenomegaly	Clumsy, dysarthria	M	c.3182T>C, c.3182T>C	p.I1061T, p.I1061T
0	3.5	2.0	16.0	Hepatosplenomegaly, jaundice	Vertical gaze palsy	M	c.3182T>C, c.3182T>C	p.I1061T, p.I1061T
0	8	1.2	12.2	Splenomegaly	Learning disability, fine motor ataxia	F*	c.3107C>T, c.2861C>T	p.T1036M, p.S954L
0.01	7	2.0	32.9	Jaundice	Vertical gaze palsy	M	c.3182T>C, c.3281T>C	p.I1061T, p.I1094T
0.3	2	1.3	44.1	Hepatosplenomegaly	Clumsiness, speech delay	M	c.3439G>T, c.3742_3745delCTCA	p.G1146V, p.L1248Vfs*3
0.5	1.5	4.5	68.0	Splenomegaly	Gross motor delay	M	c.2516T>G, c.3259T>C	p.I839R, p.F1087L
0.7	2	2.5	29.5	Splenomegaly	Clumsiness, possibly vertical gaze palsy	F	c.3493G>A, c.3741_3744delACTC	p.V1165M, p.L1248Vfs*3
1	3	1.4	38.4	Splenomegaly	Fine motor limitation	M	c.3182T>C, c.3556C>G	p.I1061T, p.R1186G
1.7	1.7	6.7	26.9	Developmental delay	Gross motor delay, speech delay	F	c.57+1G>T, Unknown	(possible splicing effect), Unknown
2	2	1.0	20.9	Splenomegaly	Clumsiness	F*	c.1920delG, c.1554—1009G>A	p.H641Tfs*2, (predicted abnormal splicing)
2	2	1.7	19.8	Splenomegaly	Clumsiness	F*	c.1920delG, c.1554—1009G>A	p.H641Tfs*2, (predicted abnormal splicing)
3	3	6.7	55.7	Fine motor ataxia	Abnormal gait, fine motor skills	F	c.2979dupA, c.2103C>G	p.D994Rfs*13, p.N701K
3	3	1.5	10.5	Learning disability	Learning disability	F*	c.410C>T, c.2000C>T	p.T137M, p.S667L
3	9	1.3	10.5	Splenomegaly	Learning disability	M*	c.3107C>T, c.2861C>T	p.T1036M, p.S954L
4	5	0.9	20.4	Hepatosplenomegaly	School difficulties	F	c.3182T>C, c.3019C>G or C>T	p.I1061T, p.F1167C
5	5	1.7	21.6	Vertical gaze palsy	Vertical gaze palsy	F	c.3182T>C, c.3182T>C	p.I1061T, p.I1061T
5	5	2.0	13.5	Developmental delay	Vertical gaze palsy	F	c.2201G>T, c.2201G>T	p.S734I, p.S734I
5	10	0.8	22.0	Hepatosplenomegaly	Clumsiness, hearing loss	F	c.2474A>G, c.289_291dupTGT	p.Y825C, p.C97_P98insC
6	6	1.2	13.3	Learning disability	Learning disability	M	c.2861C>T, Unknown	p.S954L, Unknown
6	6	2.2	8.1	Seizures	Seizures	M	c.1211G>A, c.3019C>G	p.R404Q, p.P1007A
7	7	0.8	9.8	Clumsiness	Clumsiness	F*	c.1552C>T, c.2594C>T	p.R518W, p.S865L
7	7	1.3	10.4	Learning disability	Learning disability	F*	c.1552C>T, c.2594C>T	p.R518W, p.S865L
8	8	1.2	28.3	Learning delay	Learning delay, hearing loss	M	6 poss. cDNA changes, c.3662delT	p.F842L, p.F1221Sfs*21
8	8	1.4	6.5	Learning disability	Learning disability	F	c.3182T>C, c.3019C>G or C>T	p.I1061T, p.P1007A
10	10	1.0	8.5	Learning delay	Clumsiness, learning disability	F	c.1211G>A, c.2861C>T, c.1123A>G	p.R404Q, p.S954L, p.T375A
11	11	0.9	11.2	Learning disability	Learning disability	F	c.3182T>C, c.743G>T	p.I1061T, p.G248V
12	12	0.6	13.9	Unsteady gait	Abnormal gait	M	c.743 G>T, c.3410_3411insA	p.G248V, p.N1137Kfs*121
17	17	0.9	4.8	Clumsiness	Clumsiness	M	c.3019C>G or C>T, Unknown	p.P1007A, Unknown
18	18	0.7	20.8	Psychosis	Psychosis	F	c.3176G>A, c.3742_3745delCTCA	p.R1059Q, p.L1248Vfs*3
18	18	0.6	8.0	Depression	Psychiatric symptoms	F	c.3182T>C, c.2861C>T	p.I1061T, p.S954L

*Part of a sibling pair.

**Negative values indicates age of onset in utero.

^†^Subsequently received a liver transplant.

**Figure 1:**
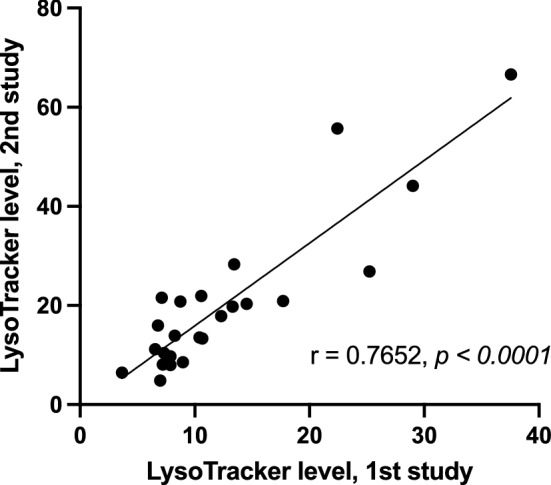
LysoTracker measurements of fibroblasts from individuals with NPC1 are reproducible. Primary fibroblast cell lines established from 24 individuals with NPC1 were previously stained with LysoTracker, and these LysoTracker levels were measured by FACS for each cell line^42^. Several years later, fibroblasts from these 24 cell lines were again subjected to the same LysoTracker staining protocol. Measurements from both studies were paired for each patient, and correlation analysis showed significant correlation of the paired measurements for these NPC1 cell lines (Spearman r = 0.7652, p < 0.0001). Line was calculated using simple linear regression.

LysoTracker levels were measured in each of the 41 fibroblast cell lines (measured as fold change over background, Table 1) using experimental methods as previously described^42^. Pairwise analysis of the LysoTracker levels for the 24 patient cell lines included in both studies showed a highly significant correlation with previous measurements (Spearman r = 0.76, *p* < 0.0001, Fig. 1), demonstrating the reproducibility of LysoTracker staining in cultured primary fibroblasts from individuals with NPC1.

### Fibroblast LysoTracker level significantly correlates with age of onset and disease severity

**Figure 2:**
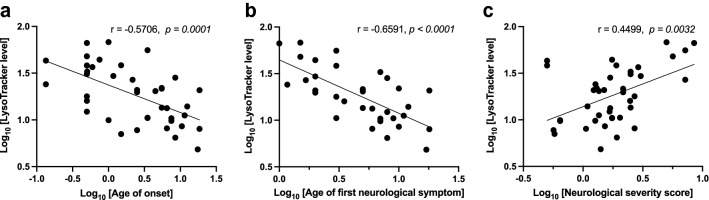
Fibroblasts from individuals with NPC1 disease show LysoTracker staining that significantly correlates with age of onset and disease severity. (**a**) LysoTracker level, measured as fold-change increase over background, is inversely correlated with the age of symptom onset. These age of onset data points included any NPC1-associated phenotypes, such as hepatosplenomegaly, jaundice and neurological abnormalities. (**b**) LysoTracker level also shows inverse correlation with the age of first neurological symptom, and this correlation is greater than that seen for any symptom onset. (**c**) LysoTracker level correlates with the age-adjusted neurological severity score (see “Materials and methods” section for score description). For all 3 graphs, each point represents a single NPC individual, and log_10_-transformed values are presented and were used for statistical analyses. P values indicate significant correlation using Spearman’s correlation; lines were calculated using simple linear regression.

Three general clinical parameters were ascertained for each individual: age of onset (including onset of any NPC1-associated symptoms such as hepatosplenomegaly, jaundice and all neurological phenotypes), age of first neurological symptom, and age-adjusted neurological severity score at the time of diagnosis (see “Materials and methods” section)^25,26^. Pairwise correlations between the 3 clinical phenotypes and LysoTracker staining levels were examined across the patient cohort, resulting in multiple significant correlations. LysoTracker level was inversely correlated with the age of onset (− 0.57, *p* = 0.0001, Fig. 2A), and showed an even greater correlation when specifically analyzing the age of first neurological symptom (− 0.66, *p* < 0.0001, Fig. 2B). In addition, LysoTracker level was directly correlated with neurological severity score (0.45, *p* = 0.0032, Fig. 2C). The significant correlations of LysoTracker levels with age of onset and neurological severity score at diagnosis suggest that LysoTracker levels could potentially serve as a valid biomarker for predicting disease onset and severity. These results confirm and extend data from the patient cohort previously described by our group^42^.

### Correlation of genome-wide expression with disease onset, severity, and cellular phenotypes

RNA was isolated from the 41 NPC1 primary fibroblast cell lines as well as parallel cultures of each cell line treated with HPβCD for 24 h. RNA sequencing (RNA-Seq) data were generated from all samples as previously described^45^. Expression levels of each gene were independently assessed for correlation with the three previously described clinical parameters (age of onset, age of first neurological symptom, and neurological severity score) and two cellular phenotypes (baseline LysoTracker level and change in LysoTracker level after HPβCD treatment, see “Materials and methods” section). These analyses identified 127 genes with expression profiles that significantly correlated with at least one clinical or cellular phenotype (Supplementary Table 1). Ten genes were correlated with age of onset, 40 genes were correlated with the age of first neurological symptom, 43 genes were correlated with neurological severity score, 21 genes were correlated with LysoTracker levels in untreated cells, and 27 genes were correlated with change in LysoTracker level following HPβCD treatment. Most genes did not overlap among these correlations, as only 14 genes correlated with two phenotypes (Supplementary Table 1). This suggests that the severity of NPC1 disease phenotypes is impacted by numerous genes/pathways rather than a small set of genes. Pathway enrichment analysis of all 127 genes aligned with the known role of NPC1 protein in cholesterol and glycosphingolipid homeostasis: the top 6 canonical pathways involved cholesterol synthesis, and the top 3 molecular and cellular functions were Lipid Metabolism, Small Molecule Biochemistry, and Vitamin and Mineral Metabolism.

The 127 significantly correlated genes could include candidate drug targets for NPC1 treatment or potential biomarkers that could be used for disease progression monitoring in diagnosed NPC1 patients. Examination of this gene list for protein biomarkers showed that *GPNMB* is the only previously suggested NPC1 disease biomarker present within the significantly correlated gene set^45,46^ (Supplementary Table 2). Additionally, biomarker analysis (Ingenuity Pathway Analysis) identified 7 of the 127 genes as highly relevant biomarkers, each of which is detectable in human blood: *GPNMB, HBEGF, HMGCR, PIK3CA, RSF1, STK11,* and *TLR4.* To identify putative drug targets, the 127 gene list was analyzed at the Drug-Gene Interaction Database (https://www.dgidb.org/)^47^. Thirty-four genes (27%) are deemed "clinically actionable" or are part of the druggable genome, and an additional 33 (26%) are in gene categories that are potentially druggable (e.g. kinases, enzymes; Supplementary Table 3). Therefore, over half (53%) of the significantly correlated genes represent potential targets for drug treatment. Additionally, genes that modify NPC1-related pathways could also be included in the significantly correlated gene set. While no lysosomal storage disorder genes were present^48^, 26/127 (20.5%) genes were associated with neurological disease in two or more patients (see functional information, Supplementary Table 1). These genes could represent novel pathways that impact neurological NPC1 disease progression.

**Figure 3:**
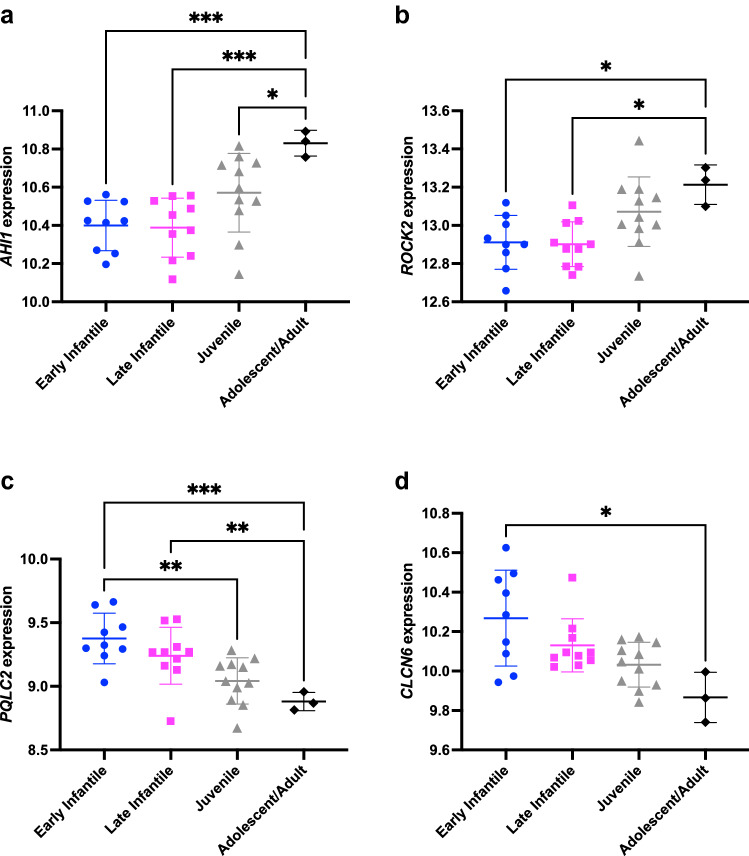
Gene expression significantly correlates with age of neurological disease onset. Four representative genes with significant correlation with the age of first neurological symptom are shown: (**a**) *AHI1*, (**b**) *ROCK2*, (**c**) *PQLC2*, and (**d**) *CLCN6*. The graphs show scatter plots of expression for each gene in each patient (variance stabilized read counts, y-axis) plotted against the age of first neurological symptom for each patient (N = 33). Patients are divided into four groups based on age of onset categories that have been previously described^86^, as follows: early infantile (2 months–2 years, N = 9), late infantile (3–6 years, N = 10), juvenile (7–15 years, N = 11), and adolescent/adulthood (> 15 years, N = 3). *AHI1* and *ROCK2* show direct correlation with age of first neurological symptom, while *PQLC2* and *CLCN6* show inverse correlation. Gene expression values are significantly different across age groups, as calculated by Welch ANOVA (p < 0.05 for all genes) followed by Dunnett’s multiple comparisons test (p values as indicated; *p < 0.05, **p < 0.01, ***p < 0.001).

Several notable genes that were highly correlated with neurological onset showed significantly different expression between patients grouped by age of onset (Fig. 3). *AHI1* (Fig. 3A) encodes a protein that localizes to primary cilia and is mutated in the rare ciliopathy Joubert syndrome 3 (OMIM #608629), which exhibits multiple neurological phenotypes, including cerebellar vermis hypoplasia and ataxia^49–52^. In addition, *Ahi1* was previously reported to show differential expression in lobule X in comparison to lobules III and VI of mouse cerebellum in both WT and *Npc1* mutants^53^, consistent with important cerebellar functions for this gene, and of interest given the differential anterior to posterior Purkinje cell degeneration seen across cerebellar lobules in NPC1 disease^54^. *ROCK2* (Fig. 3B) encodes a serine-threonine kinase that acts downstream of Rho to control neuronal cytoskeletal changes and regulate dendrite structure and function^55,56^. ROCK2 has been proposed as a possible target for treatment of many neurological diseases, including Alzheimer disease^57,58^, and also has putative roles in regulating cholesterol transport and cholesterol synthesis via the SREBP2 pathway^59,60^. *PQLC2* (Fig. 3C; a.k.a. *SLC66A1*, *LAAT-1*) encodes a lysosomal cationic amino acid transporter which recruits a heterotrimeric signaling complex composed of C9orf72, SMCR8, and WDR41 to the lysosome. Together these proteins perform critical roles in maintaining correct amino acid levels and regulating lysosomal function in response to mTORC1 and TLR signaling^61–64^. *CLCN6* (Fig. 3D) encodes a transmembrane Cl−/H+ exchanger predominantly found on late endosomes and highly expressed in the nervous system. Heterozygous mutation of *CLCN6* is associated with a rare neurological disorder that exhibits childhood-onset neurodegeneration with hypotonia, respiratory insufficiency, and brain imaging abnormalities^65^. A missense mutation of *CLCN6* has been seen in a single patient with the early infantile epileptic encephalopathy West syndrome, and this sequence change causes autophagosome accumulation and blockage of autophagosome-lysosome fusion^66^. Additionally, a *Clcn6* mouse mutant exhibits defects in lysosomal storage and mild, slowly progressing neurological abnormalities^67,68^.

### Hierarchical clustering of genes correlated with age of first neurological symptom reveals shared gene expression patterns among subsets of NPC1 fibroblasts

**Figure 4:**
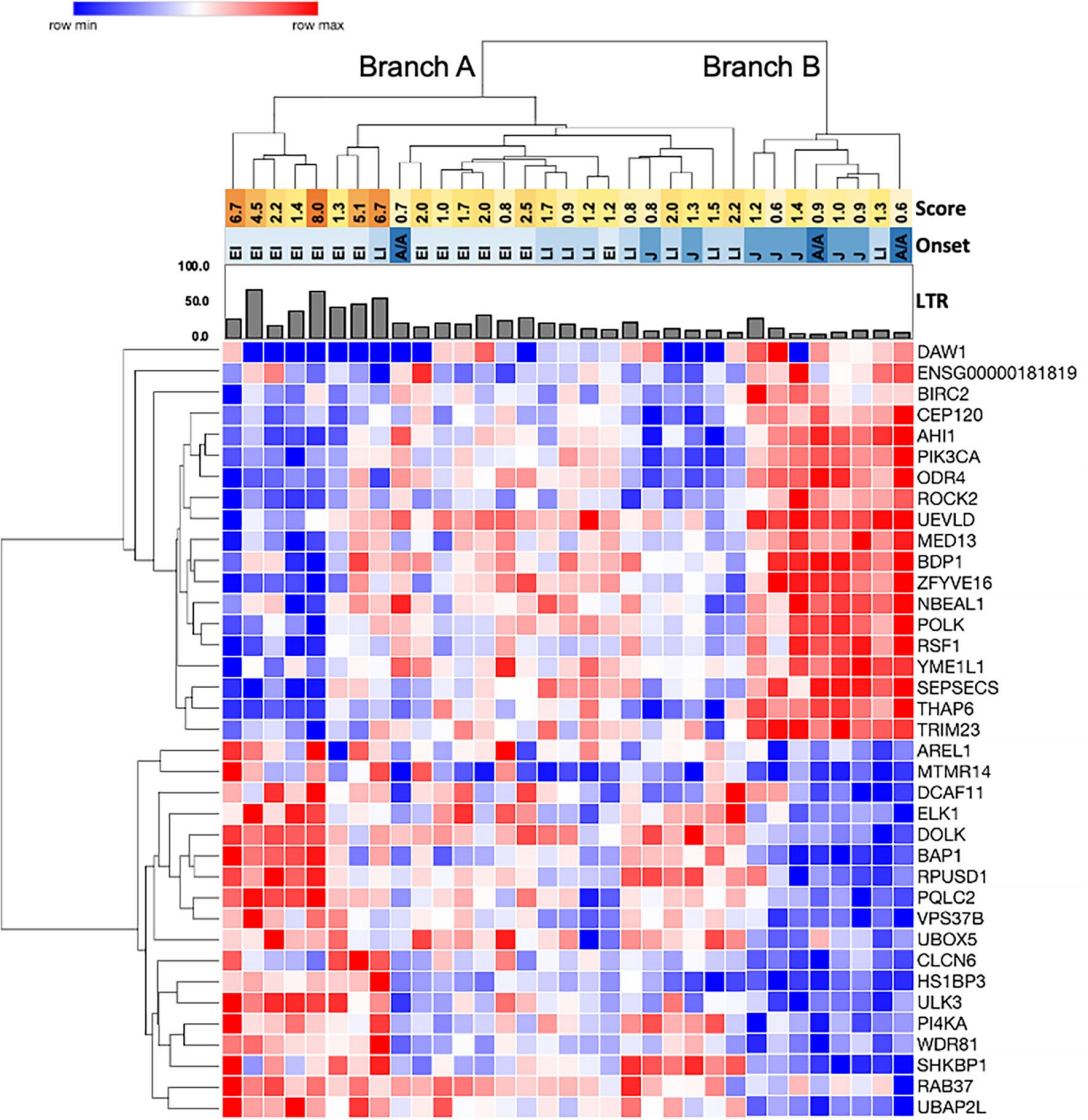
Genes correlated with age of first neurological symptom show distinct gene expression patterns among NPC1 patient subgroups. The heatmap shows hierarchical clustering of variance stabilized read counts of the 37 genes significantly correlated with the age of first neurological symptom. Patient cell lines (columns) were grouped into two high-order branches by clustering analysis, labeled Branch A and Branch B. Genes (rows) clustered into two branches, and the patient cell line Branches A and B show inverse expression of these two gene groups. Hierarchical clustering of all significantly correlated genes is shown in Supplementary Fig. 1. Score: neurological severity scores for each patient are shown in the yellow to orange heatmap, with darker colors indicating higher scores/greater severity. Onset: age of first neurological symptom is shown in the blue heatmap, with darker blues indicating older ages (EI = early infantile, LI = late infantile, J = juvenile, A/A = adolescent/adult). The bar graph indicates LysoTracker levels (LTR) in untreated fibroblasts.

Hierarchical clustering was performed using variance stabilized, normalized read counts for 121 of the 127 significantly correlated genes across the NPC1 patient cohort (six genes were removed due to low variance stabilized count levels; see Materials and methods). Despite variability across the dataset, clustering grouped distinct subsets of genes together, in particular the genes that correlated with the age of first neurological symptom or with change in LysoTracker level following HPβCD treatment (Supplementary Fig. 1). Given the clinical importance of the age of first neurological symptom, hierarchical clustering was performed using the subset of 37 genes correlated with age of first neurological symptom. This separated patient cell lines into two groups (Fig. 4, Branches A and B) and genes into two groups with relatively high or low expression in Branch B individuals (Fig. 4, rows). Interestingly, Branch B included patients with lower neurological severity scores, later disease onset, and lower LysoTracker levels. Branch A was larger and showed more variability, but it included a patient subset with the highest neurological severity scores and LysoTracker levels, all of whom had early disease onset (Fig. 4, left side of Branch A). Furthermore, this Branch A patient subset showed inverse expression of the two gene groups in comparison to Branch B. Therefore, gene expression profiles for the 37 genes correlated with the age of first neurological symptom not only revealed distinct expression patterns among subsets of patients, but also generally grouped patients by LysoTracker levels, age of first neurological symptom, and neurological severity score.

### A subset of significantly correlated genes change expression following HPβCD treatment

Animal studies and human clinical trial data indicate that HPβCD treatment may alleviate disease phenotypes in NPC1^26,28–33^. Since RNA-Seq data were collected from each patient using paired fibroblast cultures that were either untreated or treated with HPβCD, we could calculate expression changes in each gene that resulted from HPβCD treatment across the cohort (ΔHPβCD values). Hierarchical clustering using these ΔHPβCD values illustrated that most of the significantly correlated genes were not changing (Supplementary Fig. 2), suggesting that HPβCD is not affecting many potentially clinically relevant genes, including those that are correlated with the age of first neurological symptom.

**Figure 5:**
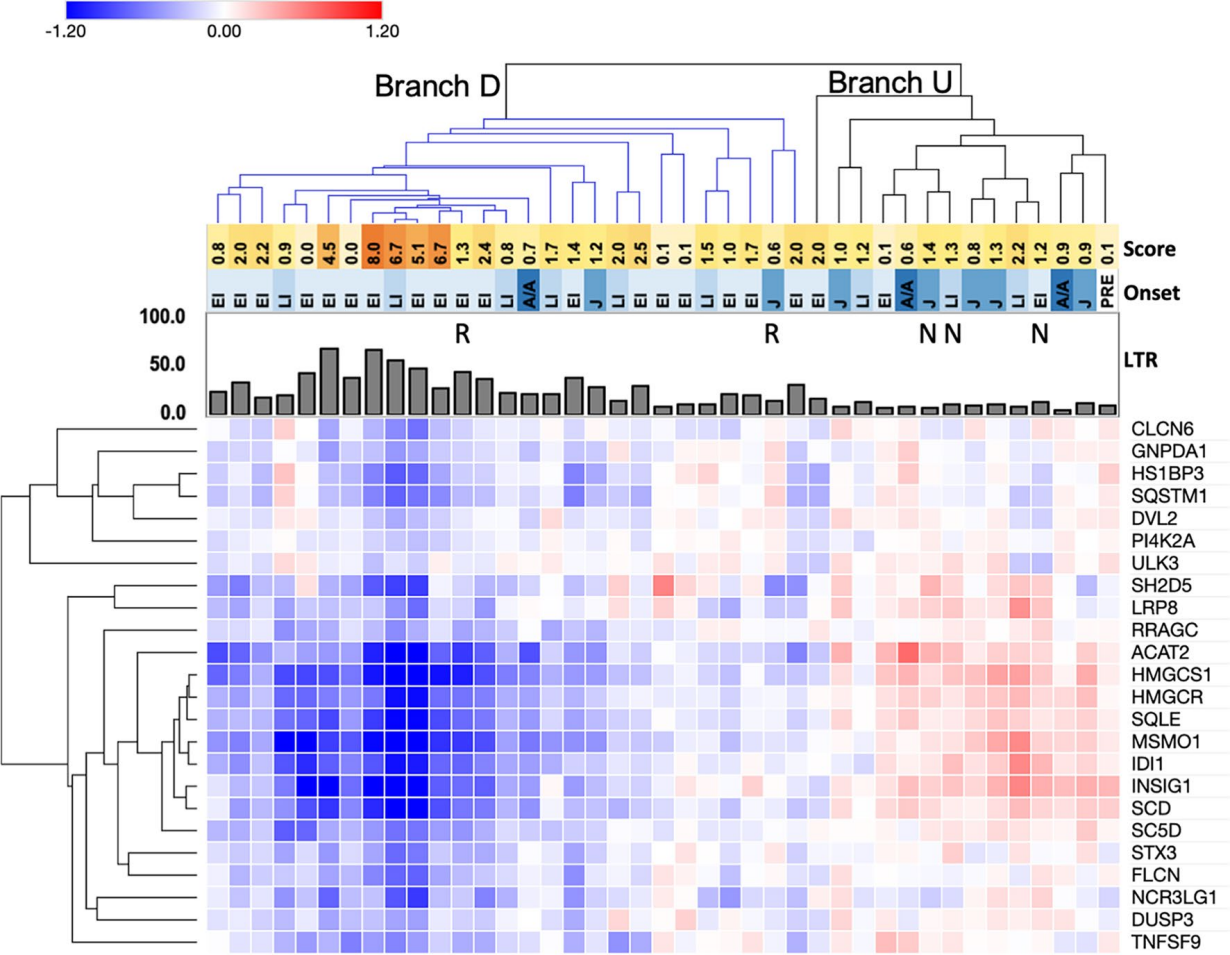
Hierarchical clustering reveals reduced expression of genes following HPβCD treatment in a subset of NPC1 patients. Heatmap values are the difference between variance stabilized read counts for cells after HPβCD treatment and for untreated cells (ΔHPβCD). Heatmap colors indicate reduced expression following HPβCD treatment (blue), unchanged expression (white), and increased expression following HPβCD treatment (red). This figure displays clustering of a subgroup of 24 genes that showed reduced expression; hierarchical clustering of ΔHPβCD values for all significantly correlated genes is shown in Supplementary Fig. 2. Branch D and Branch U are the two main dendrogram branches for patient cell lines identified by clustering analysis. Branch D (downregulated, blue color) includes cell lines with broadly reduced levels of the notable gene cluster; Branch U (upregulated/unchanged) contains the remaining cell lines with unchanged or upregulated levels of this gene cluster. R = responder, N = non-responder; these labels refer to patients who were identified in a previously reported clinical trial to show phenotypic stability/improvement in response to HPβCD treatment or no improvement, respectively^26^. Score: neurological severity scores for each patient are shown in the yellow to orange heatmap, with darker colors indicating higher scores/greater severity. Onset: age of onset (visceral or neurological) is shown in the blue heatmap, with darker blues indicating older ages (EI = early infantile, LI = late infantile, J = juvenile, A/A = adolescent/adult, PRE = presymptomatic). The bar graph indicates LysoTracker levels (LTR) in untreated fibroblasts.

However, a notable cluster of 24 genes did show expression changes, with negative ΔHPβCD values that indicated decreased expression following HPβCD treatment (Supplementary Fig. 2, Fig. 5). This cluster is enriched for genes encoding proteins involved in cholesterol synthesis and lysosomal functions. Only a subset of clustered patient cell lines showed negative ΔHPβCD values for these genes (Fig. 5, Branch D, for decreased expression), and this cluster included cell lines from patients with the highest LysoTracker levels and neurological severity scores. The remaining patient cell lines with unchanged or increased expression of these genes clustered separately (Fig. 5, Branch U, for unchanged/upregulated) and exhibited lower neurological severity scores and LysoTracker levels. Interestingly, 2 cell lines from patients who previously showed improvement in clinical parameters in response to HPβCD treatment in a clinical trial were included in Branch D (Fig. 5 and Supplementary Fig. 2, “R” labels)^26^. In contrast, 3 cell lines which clustered together in Branch U were derived from patients who did not show improvement in response to HPβCD treatment in the same study (Fig. 5 and Supplementary Fig. 2, “N” labels)^26^.

## Discussion

NPC1 is a rare disease with variable phenotypic presentation, thus necessitating unconventional approaches to gather comprehensive data from the limited number of individuals with NPC1. This study used primary fibroblasts from a cohort of NPC1 patients enrolled in an NIH natural history study to demonstrate the reproducibility of LysoTracker measurements and thus validate LysoTracker as a reliable marker of acidic cellular compartments in NPC1 cells, and to show that LysoTracker staining significantly correlates with the clinical parameters of age of onset and disease severity. Importantly, LysoTracker levels in fibroblasts, a non-neural tissue, correlate with neurological symptom onset, showing that this measure of easily accessible peripheral tissue may be a reliable indicator of neurological disease.

This study also correlated expression of specific genes with clinical age of onset, neurological disease severity, and LysoTracker levels. The significantly correlated gene set presented in this study provides potential candidates for NPC1 biomarkers, of relevance given the urgent need for biomarkers to monitor NPC1 disease progression and treatment efficacy. This dataset may also contain genes which modify NPC1 disease phenotypes. Variations in modifier genes and pathways may explain why some patients with the same *NPC1* mutation exhibit heterogeneous phenotypes and disease progression^17,24,69–71^. In support of this hypothesis, varied phenotypic severity in *Npc1* mouse models on different inbred strain backgrounds suggests the presence of modifiers, and comparative analyses between the 127 significantly correlated gene set and these studies might shed light on interesting modifier candidates^11,30,72–77^. For example, previous studies of the *Npc1*^*em1Pav*^ mouse model identified two strain-specific modifier regions, and the mouse homologs of 4 of the 127 correlated genes map to these two regions (*Slc39a10*, *Nbeal1*, *Daw1* and *Rsf1*), with two of these genes harboring strain-specific missense mutations (*Rsf1* and *Nbeal1*)^72^. Overall, the transcriptomic data in this study will provide a more comprehensive understanding of the cellular pathways perturbed by loss of NPC1 function, which may suggest new treatment avenues for NPC1 disease.

These gene expression differences reflect changes within the NPC1 patient population, and not in comparison to *NPC1*^+*/*+^ cells, which has been previously studied by other groups^78–80^. In fact, our significantly correlated gene list showed very little overlap with the genes that were previously published as significantly different from *NPC1*^+*/*+^ cells. This suggests that expression levels in NPC1 patient cells may overlap the range of expression seen in control cells, but modest expression changes could still have a modifier effect among NPC1 patients that is only revealed in the context of reduced NPC1. Indeed, moderate gene expression differences were seen across much of our significantly correlated gene set, therefore the possibility of false positives is a notable limitation of this gene set. In future studies, these genes should be independently validated using cells from a different set of NPC1 patients. This gene list provides an important initial starting point for understanding correlations of pathway and gene expression differences, and it should not be taken as a definitive, final list of candidate modifiers.

Although NPC1 individuals in this study showed heterogeneity of gene expression, hierarchical clustering of the significantly correlated genes revealed gene expression patterns that were shared among subsets of patients. The differential expression of genes associated with neurological phenotypes, including noteworthy genes with previously described roles in brain function and development (Figs. 3 and 4, Supplementary Table 1), was especially intriguing and may warrant further studies. Interestingly, the grouping of patient subsets by expression of these genes aligned with LysoTracker levels, neurological severity score, and age of onset (Fig. 4), suggesting that these gene expression differences have the potential to stratify patients into subsets based on distinct cellular pathology and disease severity. While it is premature to state that these data may represent gene signatures that would correlate with and predict NPC1 disease progression, future examination of these different expression patterns of genes and pathways and their relationship with NPC1 disease phenotypes may lead to better understanding of disease progression and facilitate discovery of new treatments.

Hierarchical clustering of the changes in gene expression after HPβCD treatment showed little to no change in most genes that correlated with clinical or cellular NPC1 phenotypes (Supplementary Fig. 2). This emphasizes the fact that current treatments such as HPβCD do not affect genes that may represent important pathways, in particular genes relevant for neurological phenotypes, and suggests that these genes/pathways may be priority novel targets for future studies on enhanced disease treatment. However, hierarchical clustering did reveal a subset of patient fibroblasts with decreased expression of a cluster of genes encoding cholesterol enzymes and lysosomal proteins. This suggests these NPC1 patient cells are responding to cholesterol redistribution, which fits with the proposed actions of HPβCD to mobilize cholesterol to the ER, restore autophagic flux, and permit secretion of lysosomal material^32,81–83^. This gene cluster also included *CLCN6, HS1BP3,* and *ULK3,* genes that were correlated with the age of neurological onset. Of note, this analysis showed these changes only occurred in some patient cell lines, and sizable differences in gene expression changes in response to HPβCD in individual patients were observed. Similar to what was seen for the genes correlated with age of neurological onset, these differences aligned to some extent with disease severity, age of onset, and cellular pathology. It is interesting to note that 2 patients previously shown as responders to HPβCD treatment^26^ showed decreased expression of this gene set, while 3 patients that previously did not show an HPβCD response showed separate clustering, with unchanged or slightly increased expression of these genes. Overall, the broad, gene expression-based view of each NPC1 patient cell line in this study provides a first glimpse at the transcriptomic complexity of patient response in the context of varied disease progression and severity, and it suggests that future studies with a larger patient cohort will be able to delve more deeply into the gene expression patterns that may underlie NPC1 disease heterogeneity.

In summary, these results provide strong evidence that fibroblast LysoTracker level may predict NPC1 disease course and severity. This information could be used to greatly facilitate disease diagnosis and management. Collection of fibroblast LysoTracker levels could result in earlier treatment as well as guide patient selection in clinical trials, identifying patients who may show a more aggressive disease progression. Furthermore, the gene set identified here suggests that differential gene expression patterns may reflect disease severity and response to therapeutic intervention, supporting future analyses to better understand these gene expression differences to help direct NPC1 treatments.

## Materials and methods

### NPC1 patient cell lines, LysoTracker staining, HPβCD treatment, and RNA-Seq analysis

Primary skin fibroblasts were obtained with written informed consent from NPC1 patients or guardians as part of a natural history study of NPC1 disease at the National Institutes of Health, which was approved by the *Eunice Kennedy Shriver* National Institute of Child Health and Human Development Institutional Review Board (NCT00344331, ClinicalTrials.gov). All methods were performed in accordance with the relevant guidelines and regulations set forth by the National Institutes of Health and the *Eunice Kennedy Shriver* National Institute of Child Health and Human Development. A variety of NPC1 mutations were present among the cohort of 41 patients used in this study (Table 1).

Fibroblast culture conditions, LysoTracker Red DND-99 staining (Invitrogen, catalog #L7528), RNA isolation, treatment with 300 µM of HPβCD (Sigma-Aldrich, Catalog no. C0926), and RNA-Seq analysis were all described in detail previously^45^. LysoTracker levels (LysoTracker fold-change, which was calculated as the ratio of the mean of LysoTracker levels in stained divided by unstained samples) were analyzed by Fluorescence-activated cell sorter (FACS) analysis, as previously published^42^. Previous studies demonstrated LysoTracker levels decrease in NPC1 cells following HPβCD treatment^45,84,85^, thus the change in LysoTracker after HPβCD treatment was calculated as the following ratio: [stained HPβCD-treated LysoTracker level minus unstained]/[stained untreated LysoTracker level minus unstained]. Duplicate biological replicates were analyzed on three different days, totaling 6 independent samples per cell line. Sequence data used for the RNA-Seq analyses are available through dbGaP, study accession number phs002392.

### RNA-seq analysis

RNA-Seq data from all samples was analyzed as previously described^45^. Briefly, reads were aligned to the GRCh38 human reference using HISAT2, reads were counted in GENCODE release 28 annotated genes using featureCounts v1.6.4, and DESeq2 v1.22.1 was used for variance-stabilized transformation, which converts counts into a log-like scale. The normalized, variance-stabilized counts were then correlated with clinical parameters and cellular phenotypes. Since there is only a single sample for each cell line, no statistical significance can be assigned to the effect on a single gene in a single cell line, therefore all counts (or differences, depending on the analysis) were collectively used to correlate to clinical parameters and cellular phenotypes.

### Statistical analyses

Pairwise Spearman’s correlations were determined among the following 5 variables for each NPC1 patient/fibroblast cell line: LysoTracker levels, change in LysoTracker levels following HPβCD treatment, age of onset (visceral or neurological), age of first neurological symptom, and age-adjusted neurological severity score at first evaluation. This score incorporates measurements of 17 clinical neurological phenotypes, and then adjusts this aggregate score to age by dividing by the age at initial evaluation^25^. A higher age-adjusted neurological severity score indicates more severe disease phenotypes.

For correlation calculations, Spearman’s rho (r_s_) values were determined for each gene in relation to each clinical parameter or cellular phenotype. Additionally, p-values were determined for the r_s_ values of each gene/covariate pair, with the Benjamini–Hochberg correction applied to control the false discovery rate (FDR). A gene was considered significantly correlated with the parameter or phenotype if the FDR of the correlation was < 0.1. Correlations and p-value corrections were performed in the R statistical programming language (version 3.5.1).

All other statistical analyses were performed using Prism software (GraphPad).

### Pathway enrichment and hierarchical clustering analysis

Pathway enrichment analysis was done using Ingenuity Pathway Analysis (Qiagen IPA). Hierarchical clustering was performed using Morpheus (https://software.broadinstitute.org/morpheus) with a distance measure of one minus Pearson’s correlation coefficient and clustering of both rows and columns. Six of the 127 genes had variance stabilized counts at the lower limit of detection in more than 50% of the patient cell lines (*ENSG00000237708*, *ENSG00000242314*, *ENSG00000255945*, *FAM153A*, *H19*, and HLA-*DQB2*). Therefore, these genes were removed from the hierarchical clustering analyses. To calculate the changes in gene expression after HPβCD treatment (ΔHPβCD) for clustering analysis, the variance stabilized count value for each gene in untreated cells was subtracted from that of HPβCD-treated cells.

## Supplementary Information

Supplementary Information.
