# Supplementary material for: Correlation of age of onset and clinical severity in Niemann–Pick disease type C1 with lysosomal abnormalities and gene expression
Source: Sci Rep. 2022 Feb 9;12:2162. doi: 10.1038/s41598-022-06112-y (PMC8828765; doi:10.1038/s41598-022-06112-y)
Supplement: Supplementary file 1 — Supplementary Information. [file 41598_2022_6112_MOESM1_ESM.pdf]

Correlation of age of onset and clinical severity in Niemann-Pick disease type C1 with lysosomal abnormalities and gene expression

Laura L. Baxter, Dawn E. Watkins-Chow, Nicholas L. Johnson, Nicole Y. Farhat, Frances M. Platt, Ryan K. Dale, Forbes D. Porter, William J. Pavan, Jorge L. Rodriguez-Gil

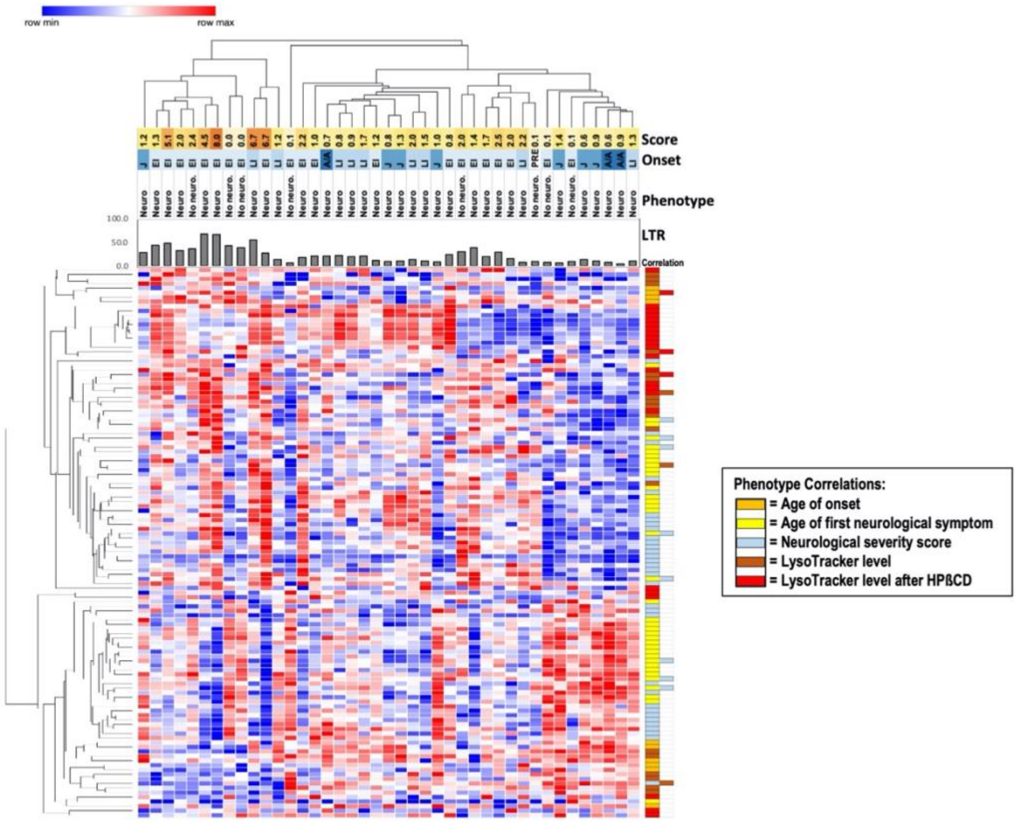

Supplementary Figure 1. Hierarchical clustering of the genes significantly correlated with age of onset, neurological severity score, or LysoTracker levels. Cell lines from NPC1 patient fibroblasts are shown in columns, and variance stabilized read count values for gene expression values for the significantly correlated genes are shown in rows. Neurological severity scores for each patient are shown in the yellow to orange heatmap, with darker colors indicating higher scores/greater severity. Age of onset is shown in the blue heatmap, with darker blues indicating older ages: EI = early infantile, LI = late infantile, J = juvenile, A/A = adolescent/adult, PRE = presymptomatic. Bar graph indicates LysoTracker (LTR) levels in untreated fibroblasts. Correlation (column to right of heatmap) indicates what clinical phenotype or cellular phenotype correlated with gene expression levels; two colored boxes for a gene represent correlation with two phenotypes. Note the clustering of genes correlated with the age of first neurological symptom (yellow correlation labels), as well as clustering of genes correlated with the change in LysoTracker levels following HPβCD treatment (red correlation labels). The genes correlated with the age of first neurological symptom are those shown in Figure 4.

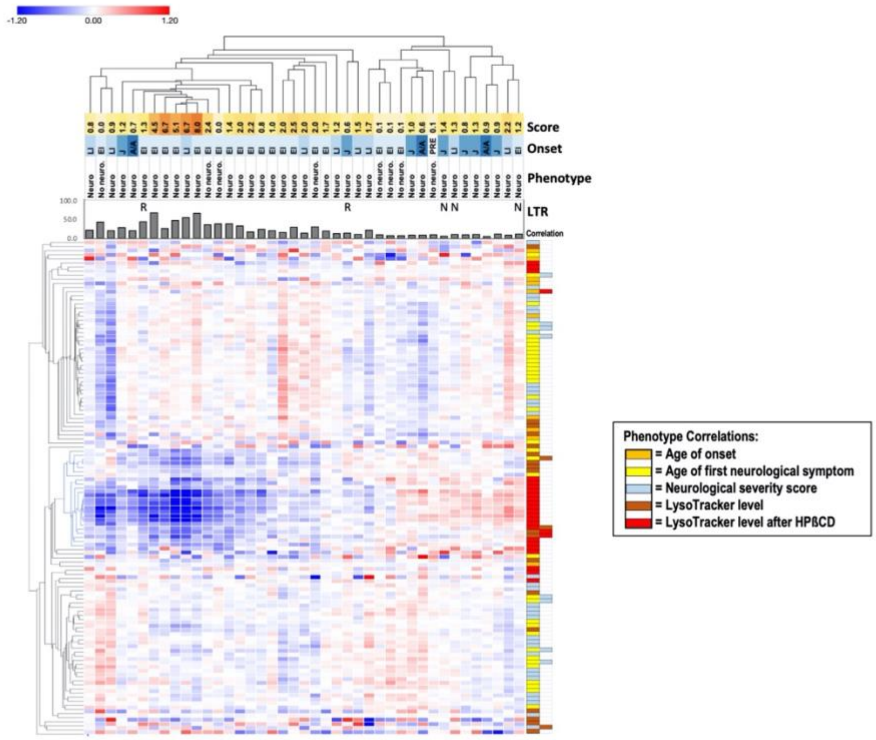

Supplementary Figure 2. Hierarchical clustering of gene expression differences seen after HPβCD treatment. The values shown are the difference between variance stabilized counts after HPβCD treatment and in untreated cells (ΔHPβCD values). Cell lines from NPC1 patient fibroblasts are shown in columns. Blue represents lower gene expression levels, reflecting a reduction in expression levels following HPβCD treatment. Neurological severity scores for each patient are shown in the yellow to orange heatmap, with darker colors indicating higher scores/greater severity. Age of onset is shown in the blue heatmap, with darker blues indicating older ages: EI = early infantile, LI = late infantile, J = juvenile, A/A = adolescent/adult, PRE = presymptomatic. Bar graph indicates LysoTracker (LTR) levels in untreated fibroblasts. Correlation (column to right of heatmap) indicates what clinical phenotype or cellular phenotype correlated with gene expression levels; two colored boxes for a gene indicate correlation with two phenotypes. Note the clustering of genes with lower expression (darker blue heatmap region, left); many of these genes are significantly correlated with the LysoTracker level following HPβCD treatment (red correlation labels). The 24 genes within this cluster (highlighted by blue brackets) are shown in Figure 5.

Supplementary Table 1. Genes which showed significant correlation of expression levels with clinical phenotypes or LysoTracker staining cellular phenotypes.

			Spearman rho values[1]					Functional information[2]	
Gene ID	Symbol	Gene description	Age of first neurological symptom	Age of onset	Age-adjusted score	LysoTracker level	LysoTracker change after HPBCD	Function	Neurological disease association
ENSG00000135541	AHI1	Abelson helper integration site 1	0.725	0.194	-0.572	-0.374	0.239	Important for cerebellar development, PMID:15322546, mutated in Joubert syndrome (also known as Jouberin), PMID:15467982; is high susceptibility gene for autism, schizophrenia, PMID:18782849; deficiency linked to depression; encodes cilia-related protein	Mutations associated with Joubert syndrome 3; OMIM #608629
ENSG00000113595	TRIM23	tripartite motif containing 23	0.687	0.420	-0.516	-0.467	0.160	Exhibits both E3 ubiquitin ligase and GTPase activities; mediates autophagy in response to viral infection, PMID:31189704; Part of innate immune system; a.k.a. ARD1	
ENSG00000134318	ROCK2	Rho associated coiled-coil containing protein kinase 2	0.680	0.288	-0.398	-0.422	0.077	Kinase that regulates cytokinesis, actin skeleton & dendrite structure/function; inhibition alters dendritic spine morphology; inhibition in Npc1-/- cells reduced growth cone collapse, PMID:20386595; specific ROCK2 inhibition enhanced neurite outgrowth of retinal ganglion cells on inhibitory substrates and induced substantial neuronal regeneration, PMID:24832597; in germinal center B cells, ROCK2 induced expression of mevalonate pathway enzymes by controlling the activity of SREBP2, the master regulator of cholesterol biosynthesis, PMID:32229726; mouse ko suggests "ROCK2 contributes to atherosclerosis, in part, by inhibiting peroxisome proliferator-activated receptor-γ-mediated reverse cholesterol transport in macrophages, which contributes to foam cell formation.," PMID:23011471; inhibition upregulates neuroprotective Parkin-mediated mitophagy, PMID:31900402; inhibition suppressed b-amyloid production in Alzheimer disease mouse model, PMID: 24305806	Proposed as possible target for Alzheimer Disease because ROCK2 inhibition increases autophagy, also proposed as general target for neurological disease treatment, PMID:33790742, 24832597; many other refs
ENSG00000121879	PIK3CA	phosphatidylinositol-4,5-bisphosphate 3-kinase catalytic subunit alpha	0.674	0.230	-0.514	-0.362	0.255	Oncogenic for a wide variety of cancers; one study suggested PIK3CA amplification in triple-negative breast cancer was associated with higher expression of cholesterol-related genes; PMID:33243007	
ENSG00000151116	UEVLD	UEV and lactate/malate dehyrogenase domains	0.658	0.259	-0.480	-0.315	0.137	Function unclear; sequence similarities to inactive E2 ubiquitin conjugating enzymes; regulates synaptogenesis in c. elegans; a.k.a. uev-3, PMID:20592265	
ENSG00000144426	NBEAL1	neurobeachin like 1	0.648	0.219	-0.481	-0.254	0.290	Golgi-associated protein, regulates cholesterol metabolism; k.d. reduces LDLR, HMGCS, and HMGCR expression, mechanism involves binding with SCAP and PAQR3 and effect on subsequent SREBP2-processing; contains BEACH domain (Beige and Chediak-Higashi); Human NBEAL1 genetic variants that exhibit reduced expression in coronary arteries are associated with coronary artery disease (PMID: 32161285)	
ENSG00000048649	RSF1	remodeling and spacing factor 1	0.640	0.264	-0.547	-0.389	0.078	Chromatin remodeling factor, also tx repressor and tx activator with Hepatitis B virus X protein	
ENSG00000039319	ZFYVE16	zinc finger FYVE-type containing 16	0.638	0.189	-0.547	-0.365	0.052	Endosomal protein (a.k.a. endofin); may regulate endosomal trafficking; facilitates TGFb signaling	
ENSG00000122008	POLK	DNA polymerase kappa	0.631	0.286	-0.499	-0.368	0.140	Specialized polymerase, which functions in DNA repair; cell lines lacking POLK show increased susceptibility to oxidative damage; associated with lung and breast cancer	
ENSG00000157181	ODR4	odr-4 GPCR localization factor homolog	0.624	0.188	-0.607	-0.360	0.001	Olfactory receptor, G-protein coupled receptor in ER, Overexpressed in lung adenocarcinoma, PMID:33251353, 24603482	
ENSG00000130600	H19	H19 imprinted maternally expressed transcript	0.623	-0.142	-0.366	0.128	0.066	Located at same genomic locus as insulin-like growth factor 2 (IGF2); long non-coding RNA that can act as tumor suppressor; Mutations iassociated with Beckwith-Wiedemann Syndrome and Wilms tumorigenesis; increased expression in serum from diabetic patients; PMID:32346662; high expression in a variety of liver diseases, PMID:33227504; suggested to play a role in regulation of angiogenesis, adipocyte differentiation, lipid metabolism, inflammatory response, cellular proliferation and apoptosis, PMID:32698876; also promotes autophagy (multiple articles)	
ENSG00000181819	ENSG00000181819	potassium channel tetramerization domain containing 9 pseudogene 2	0.619	-0.037	-0.400	-0.291	0.006	Pseudogene; a.k.a. KCTD9P2	
ENSG00000145734	BDP1	B double prime 1, subunit of RNA polymerase III transcription initiation factor IIIB	0.611	0.087	-0.423	-0.247	0.182	Encodes a subunit of the TFIIIB transcription initiation complex	
ENSG00000174796	THAP6	THAP domain containing 6	0.600	0.242	-0.548	-0.376	0.148	Function unclear	
ENSG00000109618	SEPSECS	Sep (O-phosphoserine) tRNA:Sec (selenocysteine) tRNA synthase	0.598	0.299	-0.593	-0.374	0.093	Acts in final step of synthesis of the 21st amino acid, selenocysteine (sec), which can be added at a UGA codon (termination alternate), PMID:27473727, 24987004	Associated with Pontocerebellar hypoplasia type 2D, OMIM # 613811, phenotypes typified by cerebellar and cerebral atrophy, seizures, irritability, ataxia, and extreme spasticity, PMID:27576344
ENSG00000123977	DAW1	dynein assembly factor with WD repeats 1	0.597	0.167	-0.554	-0.336	-0.025	Important for cilia motility and dynein assembly; a.k.a. ODA16	
ENSG00000242314	ENSG00000242314	ribosomal protein L12 pseudogene 32	0.596	-0.019	-0.286	-0.162	-0.300	Pseudogene, a.k.a. RPL12P32	
ENSG00000168944	CEP120	centrosomal protein 120	0.595	0.116	-0.419	-0.245	0.181	Required for centriole elongation from a procentriole, PMID:23857771	Mutation associated with Joubert syndrome 31, OMIM #617761, PMID: 27208211
ENSG00000136758	YME1L1	YME1 like 1 ATPase	0.594	0.172	-0.593	-0.353	0.269	Mitochondrial protein; part of the mTORC1-LIPIN1-YME1L axis, which regulates mitochondrial proteostasis in response to metabolic changes, such as hypoxia or nutrient starvation, PMID:31695197	
ENSG00000110330	BIRC2	baculoviral IAP repeat containing 2	0.594	0.082	-0.425	-0.247	0.295	Apoptosis inhibitor	
ENSG00000108510	MED13	mediator complex subunit 13	0.592	0.058	-0.463	-0.288	0.085	Component of the mediator complex, which acts in tx co-activation/repression; associated with glucose homeostasis, liver metabolism, and lipid metabolism, PMID: 26883362, 25422356	Mutations associated with Intellectual developmental disorder 61, OMIM #618009
ENSG00000138738	PRDM5	PR/SET domain 5	0.561	0.345	-0.649	-0.392	0.136	Tx factor, Kruppel-like zinc finger; putative tx repressor	
ENSG00000148481	MINDY3	MINDY lysine 48 deubiquitinase 3	0.543	0.209	-0.673	-0.384	0.125	Possible tumor suppressor in non-small cell lung cancer; a.k.a. C10orf97	
ENSG00000134294	SLC38A2	solute carrier family 38 member 2	0.542	0.302	-0.608	-0.469	0.208	Amino acid transporter in system A; shows sodium dependence, preference for short-chain neutral amino acids, such as alanine, serine, proline, and glutamine, as substrates, pH sensitivity, and transinhibition; regulated in response to nutritional changes, expression in liver important, and modulates systemic lipid metabolism, PMID:26268630	
ENSG00000113597	TRAPPC13	trafficking protein particle complex 13	0.532	0.346	-0.597	-0.388	0.035	Modulates autophagy and golgi stress response, k.d. reduces autophagy response, PMID:28536105; part of the TRAPPIII complex, which regulates membrane trafficking	
ENSG00000196950	SLC39A10	solute carrier family 39 member 10	0.490	0.127	-0.632	-0.383	0.270	Zinc transporter; role in immune system/macrophage function, cell division, lipid metabolism; a.k.a. ZIP10	
ENSG00000111647	UHRF1BP1L	UHRF1 binding protein 1 like	0.486	0.435	-0.660	-0.413	0.201	Binds Syntaxin 6 and has suggested role in trafficking through the early/recycling endosomal system, PMID:20163565, a.k.a SHIP164	
ENSG00000165156	ZHX1	zinc fingers and homeoboxes 1	0.476	0.304	-0.639	-0.482	0.174	Tx repressor, proposed role in cancer	
ENSG00000170074	FAM153A	family with sequence similarity 153 member A	0.459	0.161	-0.605	-0.249	-0.212	Function unclear	
ENSG00000124212	PTGIS	prostaglandin I2 synthase	0.454	0.348	-0.591	-0.597	-0.070	ER protein that catalyzes the conversion of prostglandin H2 to prostacyclin (prostaglandin I2); imbalance of prostacyclin and its antagonist thromboxane A2 contribute to the development of myocardial infarction, stroke, and atherosclerosis; upregulated expression in NPC1 patients, PMID: 31927669	
ENSG00000196967	ZNF585A	zinc finger protein 585A	0.453	0.627	-0.376	-0.365	0.109	Zinc finger protein; function unclear	
ENSG00000177234	LINC01561	long intergenic non-protein coding RNA 1561	0.449	0.278	-0.410	-0.595	-0.142	May promote non-small cell lung carcinoma and breast cancer	
ENSG00000012174	MBTPS2	membrane bound transcription factor peptidase, site 2	0.429	0.315	-0.593	-0.368	0.226	Encodes membrane-embedded zinc metalloprotease that activates signaling proteins involved in sterol control of transcription and ER stress response, PMID:23571157	Associated with X-linked IFAP syndrome with or without BRESHECK syndrome, which is linked to a quantitative decrease in cholesterol homeostasis and ER stress response, OMIM #308205
ENSG00000120370	GORAB	golgin, RAB6 interacting	0.425	0.194	-0.634	-0.295	0.136	Localized to trans-golgi;may function in vesicle tethering, based on homology to golgin family members; also binds centromere protein Sas6 to regulate centriole duplication, PMID:33704067	Associated with geroderma osteodysplastica,which shows appearance of premature aging and skin wrinkling, but also has neurological features of speech delay and mental retardation, disease also described as cutis laxa, OMIM #231070, PMID:23963297
ENSG00000162368	CMPK1	cytidine/uridine monophosphate kinase 1	0.399	0.195	-0.601	-0.273	0.231	Enzyme in nucleic acid biosynthesis	
ENSG00000136143	SUCLA2	succinate-CoA ligase ADP-forming subunit beta	0.395	0.233	-0.643	-0.289	0.264	Encodes a mitochondrial matrix enzyme that catalyzes the reversible synthesis of succinyl-CoA from succinate and CoA,	Mutation associated with a childhood-fatal encephalomyopathy, some patients show complex forms of this disorder with neurological involvement, PMID: 27651038, 33231368
ENSG00000237984	PTENP1	phosphatase and tensin homolog pseudogene 1	0.380	0.365	-0.363	-0.625	-0.061	Processed pseudogene of the tumor suppressor PTEN; can act as a tumor suppressor	
ENSG00000188641	DPYD	dihydropyrimidine dehydrogenase	0.341	0.223	-0.645	-0.246	0.111	Initial and rate-limiting enzyme in the catabolism of the pyrimidine bases uracil and thymine; mutations associated with adverse reactions to chemotherapy drugs	Mutation associated with Dihydropyrimidine dehydrogenase deficiency, which has a wide phenotypic variability, which can include neurological symptoms, OMIM #274270; possible personality disorder, bipolar disorder, major depression, and schizophrenia association, PMID: 23042115, 28632202
ENSG00000237708	ENSG00000237708	SPIN2 family pseudogene 1	0.325	-0.156	-0.598	-0.184	-0.319	Pseudogene, a.k.a. SPIN2P1	
ENSG00000152492	CCDC50	coiled-coil domain containing 50	0.298	0.594	-0.240	-0.567	0.129	Highly expressed in neuronal cells, and regulates neurite outgrowth and neuronal development, PMID: 33277610	
ENSG00000214413	BBIP1	BBSome interacting protein 1	0.294	0.610	-0.362	-0.364	-0.100	Bbsome protein, function in trafficking to cilia	Mutated in Bardet-Biedl syndrome 18, OMIM #615995, PMID:24026985, 32055034
ENSG00000081307	UBA5	ubiquitin like modifier activating enzyme 5	0.274	0.342	-0.664	-0.237	0.093	Regulates ubiquitin fold modifier pathway	Bi-allelic mutations associated with developmental and epileptic encephalopathy-44 (DEE44), OMIM #617132; other neurological disorders also reported, including a cerebellar ataxia PMID:26872069, 33853163
ENSG00000188958	UTS2B	urotensin 2B	0.264	0.668	-0.144	-0.468	-0.027	Member of the somatostatin family; encodes an 8 amino acid neuropeptide that regulates blood pressure and renal function	
ENSG00000239474	KLHL41	kelch like family member 41	0.256	0.595	-0.294	-0.362	-0.046	Substrate adaptor for E3 ubiquitin ligase Cullin-3; regulates skeletal muscle differentiation; homozygous or biallelic mutation associated with nemaline myopathy-9 (NEM9), a muscle disorder with muscle weakness, PMID:24268659	
ENSG00000264230	ANXA8L1	annexin A8 like 1	0.249	0.423	-0.428	-0.631	-0.078	Annexin family member; may function as an an anticoagulant that indirectly inhibits the thromboplastin-specific complex; overexpression associated with acute myelocytic leukemia	
ENSG00000105497	ZNF175	zinc finger protein 175	0.217	0.580	-0.430	-0.677	-0.139	A.k.a. OTK18; zinc finger protein, ubiquitious tx repressor; involved in HIV-1 replication; possible role in innate immunity, PMID: 18776638; suggested role in neuronal survival, PMID: 19247725	
ENSG00000136869	TLR4	toll like receptor 4	0.212	0.584	-0.240	-0.638	0.050	Component of the innate immune system; endosomal accumulation of TLR4 seen in NPC1 fibroblasts and NPC1-/- mice, PMID: 17314284; activated in LSD Fabry disease; PMID: 33047250, 25690728; regulates autophagy	
ENSG00000255945	ENSG00000255945	novel transcript	0.180	0.157	-0.212	-0.261	-0.572	Long non-coding RNA, function unclear	
ENSG00000182575	NXPH3	neurexophilin 3	0.177	0.195	-0.179	-0.596	0.229	Glycoprotein, highly expressed in cerebellum and cortex; important for efficient neurotransmitter release, acts as a secreted peptide	

ENSG00000159921	GNE	glucosamine (UDP-N-acetyl)-2-epimerase/N-acetylmannosamine kinase	0.119	0.079	-0.283	-0.205	0.568	Encodes the rate-limiting enzyme in the sialic acid biosynthetic pathway; GNE-associated myopathy shows cellular changes in muscle similar to AD neurological changes, PMID: 30374284	Sialuria, (which exhibits some neurological abnormalities), OMIM #269921
ENSG00000109929	SC5D	sterol-C5-desaturase	0.112	-0.057	-0.225	0.009	0.603	Cholesterol synthesis enzyme	Mutation associated with lathosterolosis (LATHOS), which includes neurological symptoms alongside other systemic abnormalities; stems from abnormal cholesterol synthesis, OMIM #607330, PMID:33204591
ENSG00000197013	ZNF429	zinc finger protein 429	0.106	0.646	-0.380	-0.412	0.173	Zinc finger protein; function unclear	
ENSG00000157110	RBPMS	RNA binding protein, mRNA processing factor	0.070	-0.058	-0.227	-0.141	-0.550	RNA binding protein with high expression in retina and heart, alternative splicing in smooth muscle, PMID: 31283468	
ENSG00000115353	TACR1	tachykinin receptor 1	0.029	0.218	-0.075	-0.001	0.577	Receptor for substance P (a.k.a. neurokinin 1); decreased mRNA levels in ataxias/dystonias, PMID: 33577922	
ENSG00000145685	LHFPL2	LHFPL tetraspan subfamily member 2	0.018	-0.605	0.021	0.362	0.554	Transmembrane protein with 4 TM domains; function unclear but mutation affects distal reproductive tract development, PMID:26964900	Variant associated with familial Parkinson disease age of onset; PMID:27402877
ENSG00000154451	GBP5	guanylate binding protein 5	-0.001	0.068	-0.147	-0.224	0.577	GTPase with a role in innate immunity and inflammation	
ENSG00000141447	OSBPL1A	oxysterol binding protein like 1A	-0.029	-0.165	0.114	0.610	0.198	Intracellular lipid receptor; heterozygous mutation associated with low HDL levels, PMID:27105157	
ENSG00000157193	LRP8	LDL receptor related protein 8	-0.052	-0.052	-0.166	-0.013	0.577	Member of LDLR family; functions as a receptor for the cholesterol transport protein ApoE; participates in transmitting Reelin signaling in cerebellar and cortical layers during development, PMID: 10380922, a.k.a. ApoER2	
ENSG00000196204	RNF216P1	ring finger protein 216 pseudogene 1	-0.094	-0.071	0.044	0.027	-0.558	Pseudogene, function unclear	
ENSG00000113070	HBEGF	heparin binding EGF like growth factor	-0.114	-0.625	0.186	0.299	0.402	Interacts with epidermal growth factor receptor (EGFR) and ERBB4; overexpression associated with metabolic diseases, PMID:32077785; cholesterol depletion induces its expression and release, PMID:21413023	
ENSG00000125657	TNFSF9	TNF superfamily member 9	-0.130	-0.141	0.179	0.307	0.576	Cytokine; immunological/Tcell activation function	
ENSG00000167202	TBC1D2B	TBC1 domain family member 2B	-0.141	-0.610	0.108	0.463	0.053	GTPAse activating protein; plays a role in vesicular trafficking, early endocytosis; recruited by RAB31 to inactivate RAB7, thus preventing multi-vesicular endosome fusion with lysosomes and allowing secretion of intraluminal vesicles, PMID:32958903	Homozygous/biallelic mutations cause Neurodevelopmental disorder with seizures and gingival overgrowth (NEDSGO), OMIM #619323
ENSG00000116954	RRAGC	Ras related GTP binding C	-0.145	-0.261	0.203	0.565	0.624	Monomeric G protein; recruits mTORC to lysosome in stress response; crucial regulator of TFEB phosphorylation by mTORC in response to amino acids, PMID:32612235, 30552228	
ENSG00000113396	SLC27A6	solute carrier family 27 member 6	-0.146	-0.612	0.168	0.365	0.059	Fatty acid transport protein; also acts as liver-specific acyl-CoA synthetase involved in uptake of long-chain fatty acids; may regulate fatty acid metabolism in mammary tissue, PMID:34100479, is downstream of SREBP-1, PMID:29846631; mutation of drosophila ortholog causes neurodegeneration, PMID:26893370	
ENSG00000184588	PDE4B	phosphodiesterase 4B	-0.195	-0.073	0.187	0.114	0.625	PDEs break down cyclic nucleotides, plays pivotal role in brain signaling, PMID: 32438615	
ENSG00000224885	ENSG00000224885	EIPR1 intronic transcript 1	-0.218	-0.325	0.076	0.632	0.235	Intronic transcript, lncRNA, function unclear, a.k.a. EIPR1-IT1	
ENSG00000233762	ENSG00000233762	ribosomal protein S15 pseudogene 4	-0.218	-0.185	0.111	0.193	-0.558	Pseudogene	
ENSG00000136235	GPNMB	glycoprotein nmb	-0.219	-0.448	0.248	0.647	0.107	Potential NPC1 biomarker; upregulated in NPC patients and mouse models, downregulated with HPBCD treatment, PMID:26771826, 33466390, 34296265	
ENSG00000171456	ASXL1	ASXL transcriptional regulator 1	-0.242	-0.118	0.607	0.232	-0.055	Chromatin binding protein; regulates adipogenesis, PMID: 21047783	Heterozygous mutation associated with Bohring-Opitz syndrome, which has profound mental retardation along with other neurological symptoms, OMIM #605039
ENSG00000249803	ENSG00000249803	novel transcript	-0.256	-0.459	0.239	0.620	-0.108	Long non-coding RNA, function unclear	
ENSG00000233250	ENSG00000233250	novel transcript	-0.263	-0.120	0.185	0.378	0.594	Long non-coding RNA, function unclear	
ENSG00000099194	SCD	stearoyl-CoA desaturase	-0.276	-0.091	0.118	0.169	0.589	Enzyme in unsaturated fatty acid biosynthesis, primarily oleic acid; important for normal autophagy, PMID: 26293158	
ENSG00000052802	MSMO1	methylsterol monooxygenase 1	-0.286	-0.023	0.024	0.239	0.621	Cholesterol synthesis enzyme	
ENSG00000113161	HMGCR	3-hydroxy-3-methylglutaryl-CoA reductase	-0.287	-0.004	0.064	0.250	0.581	Cholesterol synthesis enzyme	
ENSG00000067064	IDI1	isopentenyl-diphosphate delta isomerase 1	-0.315	-0.003	0.024	0.227	0.626	Cholesterol synthesis enzyme	
ENSG00000239857	GET4	guided entry of tail-anchored proteins factor 4	-0.325	-0.099	0.604	0.385	-0.120	Part of the transmembrane domain recognition complex (TRC), which targets cytoplasmic C-terminal tail-anchored proteins to ER, Golgi, and mitochondrial membranes	
ENSG00000120437	ACAT2	acetyl-CoA acetyltransferase 2	-0.331	-0.014	0.107	0.225	0.552	Cholesterol synthesis enzyme	
ENSG00000204160	ZDHHC18	zinc finger DHHC-type palmitoyltransferase 18	-0.331	-0.300	0.591	0.402	0.164	Golgi-resident palmitoyl transferase	
ENSG00000225190	PLEKHM1	pleckstrin homology and RUN domain containing M1	-0.348	-0.384	0.426	0.605	0.061	Acts as endolysosomal adaptor to mediate autophagosome/lysosome fusion, regulated by MTOR, PMID:33452816; also regulated by PI4P-Rab7 pathway (PI4P produced by PI4K2A, another sig. gene), PMID:31368593	
ENSG00000054116	TRAPPC3	trafficking protein particle complex 3	-0.364	-0.343	0.382	0.599	0.045	Part of the TRAPP complex, which tethers transport vesicles to the cis-Golgi membrane; specifically regulates transport from ER to golgi, PMID:15728249	
ENSG00000112972	HMGCS1	3-hydroxy-3-methylglutaryl-CoA synthase 1	-0.372	-0.010	0.101	0.239	0.617	Cholesterol synthesis enzyme	
ENSG00000171302	CANT1	calcium activated nucleotidase 1	-0.375	-0.149	0.628	0.403	0.040	Function unclear, member of the apyrase family, which are calcium-activated, plasma membrane-bound enzymes; highly expressed in clear cell renal cell carcinoma, PMID: 31102300	Mutation associated with Desbuquois dysplasia 1, which involves numerous skeletal abnormalities as well as some neurological abnormalites, OMIM #251450
ENSG00000104549	SQLE	squalene epoxidase	-0.384	-0.067	0.112	0.244	0.586	Cholesterol synthesis enzyme; catalyzes the first oxygenation step in sterol biosynthesis and is thought to be one of the rate-limiting enzymes in this pathway	
ENSG00000188211	NCR3LG1	natural killer cell cytotoxicity receptor 3 ligand 1	-0.385	-0.331	0.237	0.591	0.556	Expressed on tumor cells; elicits natural killer cell cytotoxicity	
ENSG00000166900	STX3	syntaxin 3	-0.398	-0.368	0.485	0.667	0.626	Regulates vesicle fusion and endocytosis associated with trafficking	Reports of two families with STX3 mutations and neurological phenotypes; PMID:30909251, 25358429
ENSG00000225094	ENSG00000225094	SET pseudogene 20	-0.405	-0.200	0.609	0.200	-0.023	Pseudogene, a.k.a. SETP20	
ENSG00000154803	FLCN	folliculin	-0.410	-0.466	0.395	0.750	0.637	Regulates lysosomal positioning, nutrient sensing, and mTORC signaling to lysosomes; mutated in Birt-Hogg-Dubé (BHD) syndrome, PMID:33981707, 32612235, 32195250, 31672913	
ENSG00000113552	GNPDA1	glucosamine-6-phosphate deaminase 1	-0.421	-0.394	0.447	0.731	0.235	Part of carbohydrate metabolic pathway	
ENSG00000125386	FAM193A	family with sequence similarity 193 member A	-0.432	-0.204	0.630	0.304	-0.166	Binds and regulates PA28γ, which is a 20S proeaosome activator, PMID:29934401	
ENSG00000177732	SOX12	SRY-box transcription factor 12	-0.436	-0.072	0.613	0.365	-0.167	SRY box tx factor, SOX C group, promotes cell fate determination, PMID:26830765, promotes cancer progression and metastasis in various cancers	
ENSG00000259956	RBM15B	RNA binding motif protein 15B	-0.440	-0.482	0.592	0.459	-0.176	RNA binding motif protein with post-transcriptional regulatory activity; binds splicing factor compartment and the nuclear envelope as well as mRNA export factors NXF1 and Aly/REF, PMID:19586903; mediates XIST-directed transcriptional silencing, PMID:27602518	
ENSG00000189410	SH2D5	SH2 domain containing 5	-0.446	-0.348	0.501	0.509	0.588	Function unclear; enriched in brain, particularly cerebellar Purkinje cells; may regulate Rac1-GTP levels, PMID: 25331951	
ENSG00000155252	PI4K2A	phosphatidylinositol 4-kinase type 2 alpha	-0.451	-0.491	0.424	0.681	0.219	Phosphorylates PtdIns at the D-4 position, an essential step in the biosynthesis of Phosphatidylinositolpolyphosphates (PtdInsPs); modulates phosphoinositide levels at autolysosomes; accumulates in mouse models for hereditary spastic paraplegia; regulates autophagosome/lysosome fusion; PMID: 31368593, 33618608	Homozygous nonsense mutation in one family associated with disorder showing global developmental delay, dystonia, disturbed sleep, and heat intolerance; another patient with homozygous missense mutation showed neurological phenotype and cutis laxa, PMID:32418222; mouse model shows neurological phenotype that includes cerebellar gliosis and Purkinje cell loss, PMID:30564627, 19581584
ENSG00000118046	STK11	serine/threonine kinase 11	-0.452	-0.151	0.648	0.343	-0.351	"Master protein kinase," regulates cell polarity and energy metabolism, functioning to inhibit liver gluconeogenesis, PMID: 33731695; induces autophagy via AKB1, PMID: 29480489, 28561066 and many other refs, a.k.a. Lkb1	
ENSG00000186480	INSIG1	insulin induced gene 1	-0.454	-0.089	0.147	0.290	0.594	Regulates cholesterol metabolism/homeostasis, lipogenesis, and glucose homeostasis; binds SCAP and facilitates retention of the SCAP/SREBP complex in the ER	
ENSG00000166166	TRMT61A	tRNA methyltransferase 61A	-0.462	-0.301	0.634	0.418	-0.063	Functions in tRNA processing	
ENSG00000004975	DVL2	dishevelled segment polarity protein 2	-0.465	-0.381	0.380	0.614	0.121	Involved in Wnt signaling pathway	
ENSG00000136295	TTYH3	tweety family member 3	-0.476	-0.219	0.616	0.462	-0.127	Encodes large-conductance chloride channel, calcium dependent, so may regulate Ca2+ signaling, PMID: 15010458; widely expressed, with strong neuronal expression in early development, PMID: 34262434	
ENSG00000204713	TRIM27	tripartite motif containing 27	-0.476	-0.290	0.689	0.347	-0.047	E3 ubiquitin ligase that partners with USP7	
ENSG00000161011	SQSTM1	sequestosome 1	-0.489	-0.463	0.547	0.610	0.321	Critical autophagy regulatory protein; involved in mTOR signaling, cholesterol efflux under stress, nutrient sensing, inflammation, PMID:30499183, a.k.a. p62	Mutation associated with Frontotemporal dementia and/or amyotrophic lateral sclerosis 3 OMIM #616437; Neurodegeneration with ataxia, dystonia, and gaze palsy, childhood-onset, OMIM #617145
ENSG00000160209	PDXK	pyridoxal kinase	-0.489	-0.224	0.601	0.320	0.041	Acts to phosphorylate Vitamin B6	Biallelic mutations associated with primary axonal polyneuropathy and optic atrophy, PMID: 31187503, OMIM #618511
ENSG00000076201	PTPN23	protein tyrosine phosphatase non-receptor type 23	-0.494	-0.218	0.607	0.474	-0.085	Member of ESCRT (Endosomal Sorting Complexes Required for Transport); acts as tumor suppressor; also associated with ciliogenesis and RNA splicing, PMID: 31395947; regulates synaptic neuropeptide release, PMID:29378961	Mutation associated with Neurodevelopmental disorder and structural brain anomalies with or without seizures and spasticity, OMIM #618890, PMID: 31395947
ENSG00000108861	DUSP3	dual specificity phosphatase 3	-0.514	-0.225	0.273	0.263	0.577	Acts to dephosphorylate threonine/serine and tyrosine residues, known substrates include the MAPKs ERK and JNK, STAT5 and ErbB29; Dusp3 KO mice suggest it plays a role in obesity, insulin resistance, NAFLD and liver damage, PMID:33712680	
ENSG00000138867	GUCD1	guanylyl cyclase domain containing 1	-0.537	-0.243	0.660	0.481	-0.050	Converts GTP to cGMP, interacts with the E3 Ubiquitin ligase NEDD4, regulates liver regeneration, PMID: 24743017	
ENSG00000269190	FBXO17	F-box protein 17	-0.546	-0.178	0.600	0.192	-0.145	Part of SCF complexes (formed by SKP1, cullin (CUL1), and F-box proteins), which act as protein-ubiquitin ligases; promotes cell proliferation, activates Akt, Wnt-bcatenin pathway	
ENSG00000205903	ZNF316	zinc finger protein 316	-0.546	-0.245	0.657	0.525	0.085	Zinc finger protein; function unclear	
ENSG00000107263	RAPGEF1	Rap guanine nucleotide exchange factor 1	-0.548	-0.262	0.641	0.364	-0.099	Adaptor protein, ubiquitous, essential for normal development; promotes ERK1/2 phosphorylation, involved in cell adhesion and neuronal migration, neural precursor population size, neuronal migration, PMID:16858399, 18506028, a.k.a. C3G	Two patients from consanguineous family with homozygous mutations and neuropsychiatric disorder, PMID:33834495
ENSG00000135632	SMYD5	SMYD family member 5	-0.582	-0.304	0.650	0.337	-0.203	Regulates heterochromatin and chromosome integrity during ES cell differentiation, PMID: 28951459; contains SET and MYND domains for lysine methylation and protein-protein interaction, respectively	
ENSG00000232629	HLA-DQB2	major histocompatibility complex, class II, DQ beta 2	-0.593	-0.438	0.324	0.274	0.090	Part of the major histocompatibility complex	
ENSG00000140474	ULK3	unc-51 like kinase 3	-0.595	-0.515	0.455	0.589	0.070	Interacts with ESCRT complexes, governing the timing of membrane abscission in exosome budding and cytokinesis, PMID:34190988; also regulates autophagy, PMID:33988680, 27717182	
ENSG00000185019	UBOX5	U-box domain containing 5	-0.598	-0.190	0.295	0.112	-0.138	Encodes lncRNA, involved in ubiquitin-mediated proteolysis, upregulated in endometriosis	

ENSG00000119682	AREL1	apoptosis resistant E3 ubiquitin protein ligase 1	-0.600	-0.249	0.621	0.316	0.323	E3 ubiquitin ligase that limits cellular response to apoptosis	
ENSG00000163719	MTMR14	myotubularin related protein 14	-0.600	-0.315	0.577	0.333	-0.243	Muscle-specific inositide phosphatase, regulates muscle cell calcium homeostasis; regulates autophagy, KO mice fed a high fat diet showed greater obesity, lipid accumulation/dysfunction, and inflammation; PMID:27807764, 26697164	
ENSG00000172794	RAB37	RAB37, member RAS oncogene family	-0.604	-0.218	0.395	0.301	0.322	GTPase which regulates vesicular trafficking; promotes autophagosome accumulation and regulates autophagosome biogenesis, PMID: 29229996, 29388490	
ENSG00000007376	RPUSD1	RNA pseudouridine synthase domain containing 1	-0.604	-0.163	0.529	0.386	-0.057	RNA binding protein, putative pseudouridine synthase activity	
ENSG00000118960	HS1BP3	HCLS1 binding protein 3	-0.604	-0.490	0.485	0.732	0.268	Autophagy inhibitor, PMID:28318354	
ENSG00000040487	PQLC2	solute carrier family 66 member 1	-0.614	-0.343	0.697	0.567	0.065	Official name = SLC66A1, a.k.a. LAAT-1; Lysosomal cationic amino acid transporter, transports lysine and arginine at the lysosome, and knockdown causes enlarged lysosomes in C. elegans, PMID: 22822152, 23169667; recruits the C9orf72-SMCR8 - WDR41 complex to lysosomes when amino acids are scarce, PMID:31851326, 33597295	
ENSG00000126767	ELK1	ETS transcription factor ELK1	-0.617	-0.314	0.378	0.323	-0.203	Tx factor with role downstream of EGFR and P38/Erk2 signaling; along with c-Jun N-terminal kinase (JNK) , plays role in HSP70-induced downregulation of ABCA1 and ABCG1; k.d. promotes atherosclerosis; PMID:29678642	
ENSG00000160410	SHKBP1	SH3KBP1 binding protein 1	-0.617	0.011	0.484	0.283	-0.043	Member of the potassium channel tetramerization domain (KCTD) family of E3 ligases; acts upstream of EGFR to prevent its degradation; Shkbp1 ko mice show reduced tumors in comparison to wt when chemically induced to form tumors, PMID:34112919	
ENSG00000241973	PI4KA	phosphatidylinositol 4-kinase alpha	-0.617	-0.111	0.452	0.304	-0.103	Encodes phosphatidylinositol (PI) 4-kinase; generates the lipid signalling phosphoinositides, which act as signalling enzymes that control numerous aspects of cellular response to the extracellular environment; also aid in regulating membrane trafficking and lipid transport	
ENSG00000139722	VPS37B	VPS37B subunit of ESCRT-I	-0.619	-0.441	0.452	0.520	0.133	Part of endosomal sorting complex (ESCRT-1), which performs roles in autophagosome closure, multivesicular body biogenesis, and cytokinesis; knockdown in cell culture causes cell stress response; PMID:33419951	
ENSG00000011021	CLCN6	chloride voltage-gated channel 6	-0.620	-0.189	0.310	0.399	0.184	Encodes a transmembrane Cl-/H+ exchanger on late endosomes; modulates luminal ion composition; expressed in nervous system; expression of human mutation caused autophagosome accumulation and impaired the clearance of autophagosomes by blocking autophagosome-lysosome fusion, PMID:33590434; mutations in mouse caused phenotypes consistent with lysosomal storage abnormalities as well as a slowly progressing neurological phenotype; PMID:16950870, 21107136	Childhood-onset neurodegeneration with hypotonia, respiratory insufficiency, and brain imaging abnormalities (CONRIBA) is caused by heterozygous mutation, OMIM #619173
ENSG00000163930	BAP1	BRCA1 associated protein 1	-0.623	-0.248	0.609	0.458	0.009	Encodes nuclear ubiquitin carboxy-terminal hydrolase / deubiquitinase; upstream regulator of mTOR signaling, PMID:32819560; downregulation in inducible mouse model was associated with increased cholesterol biosynthesis machinery and reduced expression of gluconeogenic and lipid homeostasis proteins in liver, PMID:27373151; inhibits autophagy in melanoma cells, PMID:33516665	
ENSG00000167716	WDR81	WD repeat domain 81	-0.626	-0.287	0.541	0.467	0.110	Interacts with WDR91 as part of an endosomal protein complex that inhibits PI3 Kinase, which permits loss of PtdIns3P and allows conversion of early to late endosomes, PMID: 26975852; important for late endosome fusion, PMID:27126989; also important role in autophagy, PMID:28404643	Cerebellar ataxia, mental retardation, and dysequilibrium syndrome 2, OMIM #610185; Hydrocephalus, congenital, 3, with brain anomalies, OMIM #617967; mice homozygous for a Wdr81 missense mutation show progressive ataxia along with Purkinje cell loss, PMID: 23595742; also regulates adult hippocampal neurogenesis, PMID: 30531936
ENSG00000100897	DCAF11	DDB1 and CUL4 associated factor 11	-0.631	-0.170	0.594	0.374	-0.071	E3 ubiquitin ligase; acts as E3 ligase substrate adaptor, mediating degradation of a variety of substrates, a.k.a. WDR23	
ENSG00000143569	UBAP2L	ubiquitin associated protein 2 like	-0.656	-0.335	0.645	0.529	-0.060	RNA binding protein; tethering function enhances translation PMID: 32807991	
ENSG00000175283	DOLK	dolichol kinase	-0.678	-0.170	0.480	0.343	0.039	Encodes the enzyme responsible for final step of the de novo biosynthesis of dolichol phosphate; involved in processes for protein glycosylation and GPI anchors in the ER	Mutation associated with congenital disorder of glycosylation, type Im, OMIM# 610768; most common features are epilepsy, intellectual disability, myopathies, neuropathies and stroke-like episodes; in some patients, hypotonia, seizures, hypsarrhythmia; also regulates startle magnitude in zebrafish, PMID:34061829

[1]Rho values with significant p values ≤0.1 are shown in red and green for negative or positive correlations, respectively

[2]Function found at NCBI gene and from 5 PubMed searches: gene name + lysosome, cholesterol, lipids, NPC, and autophagy; Neurological disease association searches involved OMIM and PubMed searches

Supplementary Table 2. ***GPNMB*** **is the only previously proposed protein biomarker with significant correlation of gene expression to clinical parameters in this NPC1 patient cohort.**

Ensembl gene ID	Symbol	LysoTracker levels_rho (P value)	Age of first neurological symptom_rho (P value)	Age of onset_rho (P value)	Neurological severity score Score_rho (P value)	DeltaLTR_ rho (P value)
ENSG00000104327	***CALB1***	-0.3432 (0.5548)	0.4445 (0.3386)	0.3307 (0.5779)	-0.0929 (0.9101)	-0.1398 (0.8867)
ENSG00000277632	***CCL3***	-0.3202 (0.5979)	0.0962 (0.9060)	0.2080 (0.7510)	-0.2088 (0.7500)	0.3128 (0.6980)
ENSG00000164733	***CTSB***	0.1420 (0.8448)	-0.0434 (0.9639)	-0.2923 (0.6542)	0.0795 (0.9258)	-0.1795 (0.8412)
ENSG00000117984	***CTSD***	0.3045 (0.6298)	-0.2773 (0.6833)	-0.2459 (0.7051)	0.1761 (0.7949)	-0.0824 (0.9417)
ENSG00000163131	***CTSS***	0.1293 (0.8618)	-0.0814 (0.9226)	0.0574 (0.9499)	-0.0407 (0.9676)	-0.0540 (0.9656)
ENSG00000121769	***FABP3***	0.3370 (0.5663)	-0.2422 (0.7068)	-0.3274 (0.5834)	0.1170 (0.8768)	0.4039 (0.4486)
ENSG00000136235	***GPNMB***	**0.6465 (0.0424)**	-0.2191 (0.7364)	-0.4482 (0.3310)	0.2485 (0.7022)	0.1073 (0.9200)
ENSG00000243955	***GSTA1***	-0.1407 (0.8477)	0.2057 (0.7510)	0.1039 (0.8938)	0.0261 (0.9805)	0.0129 (0.9939)
ENSG00000170606	***HSPA4***	0.1353 (0.8569)	0.1401 (0.8490)	-0.1345 (0.8569)	-0.1264 (0.8663)	0.1338 (0.8912)
ENSG00000131981	***LGALS3***	0.1317 (0.8570)	-0.0391 (0.9695)	0.1727 (0.7965)	-0.0270 (0.9791)	-0.1216 (0.9044)
ENSG00000090382	***LYZ***	NA	NA	NA	NA	NA
ENSG00000186868	***MAPT***	0.1642 (0.8122)	-0.1342 (0.8569)	-0.2834 (0.6710)	0.0013 (0.9988)	-0.0056 (0.9950)
ENSG00000142168	***SOD1***	-0.1257 (0.8663)	-0.1753 (0.7949)	0.1363 (0.8564)	-0.0917 (0.9101)	-0.0992 (0.9270)

Supplementary Table 3. Drug target candidates within the significantly correlated gene list.

Gene	Category	Sources	Number of drug interactions
ASXL1	CLINICALLY ACTIONABLE	MskImpact|FoundationOneGenes|CarisMolecularIntelligence|Tempus	2
BAP1	CLINICALLY ACTIONABLE	MskImpact|FoundationOneGenes|CarisMolecularIntelligence|Oncomine|Tempus	6
CANT1	CLINICALLY ACTIONABLE	CarisMolecularIntelligence	
DPYD	CLINICALLY ACTIONABLE and DRUGGABLE GENOME	Tempus|CIViC and HingoraniCasas|RussLampel	9
FLCN	CLINICALLY ACTIONABLE	MskImpact|FoundationOneGenes|CarisMolecularIntelligence|Tempus	2
H19	CLINICALLY ACTIONABLE	Tempus	
HLA-DQB2	CLINICALLY ACTIONABLE	Tempus	
PIK3CA	CLINICALLY ACTIONABLE and DRUGGABLE GENOME	MskImpact|FoundationOneGenes|CarisMolecularIntelligence|Oncomine|Tempus |CIViC and HopkinsGroom|RussLampel|HingoraniCasas	152
RSF1	CLINICALLY ACTIONABLE	Tempus	
STK11	CLINICALLY ACTIONABLE and DRUGGABLE GENOME	MskImpact|FoundationOneGenes|CarisMolecularIntelligence|Oncomine|Tempus and RussLampel|HingoraniCasas|HopkinsGroom	23
TRIM27	CLINICALLY ACTIONABLE and DRUGGABLE GENOME	CarisMolecularIntelligence and HopkinsGroom	
BIRC2	DRUGGABLE GENOME	HingoraniCasas	7
CLCN6	DRUGGABLE GENOME	HopkinsGroom|RussLampel	
DUSP3	DRUGGABLE GENOME	HingoraniCasas|RussLampel|HopkinsGroom	7
GPNMB	DRUGGABLE GENOME	HingoraniCasas	1
HBEGF	DRUGGABLE GENOME	HingoraniCasas	3
HMGCR	DRUGGABLE GENOME	RussLampel|HopkinsGroom|HingoraniCasas	19
HMGCS1	DRUGGABLE GENOME	RussLampel|HopkinsGroom	
LRP8	DRUGGABLE GENOME	HingoraniCasas	
NXPH3	DRUGGABLE GENOME	HingoraniCasas	
ODR4	DRUGGABLE GENOME	HopkinsGroom	
PDE4B	DRUGGABLE GENOME	HingoraniCasas|RussLampel|HopkinsGroom	33
PDXK	DRUGGABLE GENOME	RussLampel	
PI4K2A	DRUGGABLE GENOME	HopkinsGroom	1
PI4KA	DRUGGABLE GENOME	HopkinsGroom|RussLampel	2
PTGIS	DRUGGABLE GENOME	HopkinsGroom|RussLampel	1
PTPN23	DRUGGABLE GENOME	HopkinsGroom|RussLampel	
ROCK2	DRUGGABLE GENOME	RussLampel|HopkinsGroom|HingoraniCasas	34
SCD	DRUGGABLE GENOME	HingoraniCasas	5
SQLE	DRUGGABLE GENOME	HingoraniCasas|RussLampel|HopkinsGroom	3
TACR1	DRUGGABLE GENOME	HingoraniCasas|HopkinsGroom|RussLampel	36
TLR4	DRUGGABLE GENOME	HingoraniCasas	11
ULK3	DRUGGABLE GENOME	HopkinsGroom|RussLampel|HingoraniCasas	2
UTS2B	DRUGGABLE GENOME	HingoraniCasas	
ACAT2	ENZYME	GuideToPharmacology|Pharos	
AREL1	ENZYME	Pharos	
GNPDA1	ENZYME	Pharos	
IDI1	ENZYME	GuideToPharmacology|Pharos	
MSMO1	ENZYME	HumanProteinAtlas	
POLK	ENZYME	Pharos	263
SC5D	ENZYME	HumanProteinAtlas|Pharos	
SEPSECS	ENZYME	HumanProteinAtlas	
SUCLA2	ENZYME	HumanProteinAtlas|Pharos	
UBA5	ENZYME	Pharos	
UBOX5	ENZYME	Pharos	
UEVLD	ENZYME	Pharos	
TTYH3	ION CHANNEL	Pharos	
CMPK1	KINASE	Pharos	3
DOLK	KINASE	Pharos	
GNE	KINASE	Pharos	
GORAB	KINASE	Pharos	
TRIM23	KINASE	Pharos	
WDR81	KINASE	Pharos	
SMYD5	METHYL TRANSFERASE	BaderLabGenes	
MTMR14	MYOTUBULARIN RELATED PROTEIN PHOSPHATASE	dGene	
YME1L1	PROTEASE	dGene	
SQSTM1	SERINE THREONINE KINASE	GO	1
BDP1	TRANSCRIPTION FACTOR	Pharos	
ELK1	TRANSCRIPTION FACTOR	Pharos	
MBTPS2	TRANSCRIPTION FACTOR	Pharos	
MED13	TRANSCRIPTION FACTOR	Pharos	
PRDM5	TRANSCRIPTION FACTOR	Pharos	
SOX12	TRANSCRIPTION FACTOR	Pharos	
PQLC2	TRANSPORTER	Pharos	
SLC27A6	TRANSPORTER	GuideToPharmacology|Pharos	
SLC38A2	TRANSPORTER	GuideToPharmacology|Pharos	
SLC39A10	TRANSPORTER	GuideToPharmacology|Pharos	
